# Supplementary material for: Spironolactone for adult female acne (SAFA): protocol for a double-blind, placebo-controlled, phase III randomised study of spironolactone as systemic therapy for acne in adult women
Source: BMJ Open. 2021 Aug 26;11(8):e053876. doi: 10.1136/bmjopen-2021-053876 (PMC8395279; doi:10.1136/bmjopen-2021-053876)
Supplement: Supplementary data [file bmjopen-2021-053876supp001.pdf]

(Print on local headed paper)

REC Number: 18/WA/0420
IRAS ID: 246637

SAFA INFORMED CONSENT FORM

SAFA: Spironolactone for Adult Female Acne: pragmatic multicentre double-blind randomised superiority trial to investigate the clinical and cost-effectiveness of spironolactone for moderate or severe persistent acne in women

Participant Identification Number for this trial: | | | | | | | | |

Name of Researcher: ________________________________

Please initial each box

1.	I confirm that I have read the participant information sheet (version __ dated DD-MMM-YYYY) for the above study. I have had the opportunity to consider the information, ask questions and have had these answered satisfactorily.	INITIAL
2.	I understand that my participation is voluntary and that I am free to withdraw at any time without giving any reason and without my medical care or legal rights being adversely affected.	INITIAL
3.	I give permission for a copy of my consent form to be sent to the Southampton Clinical Trials Unit (where it will be stored securely), to allow confirmation of my consent.	INITIAL
4.	I agree for my contact details to be shared with the Southampton Clinical Trials Unit (where stored securely), to allow the study team to contact me if needed in an emergency situation (e.g. during a pandemic).	INITIAL
5.	I understand that relevant sections of my medical records, and data collected during the study, may be looked at by individuals from the Sponsor or their delegates, from Regulatory Authorities, or from the NHS Trust where it is relevant to my taking part in this research. I give permission for these individuals to have access to my records.	INITIAL
6.	I understand that the information collected about me may be used to support other ethically approved research in the future, and may be shared in a pseudonymised form with other researchers.	INITIAL
7.	I agree to my General Practitioner being informed of my participation in the study and to receiving information critical to my care.	INITIAL
8.	I agree to not donate blood during the first 6 months of the study.	INITIAL

SAFA Informed Consent Form v4 05-MAR-2021.docx

Funded by NIHR

(Print on local headed paper)

9. I agree to photographs of my facial acne being taken at the first clinic visit. INITIAL

10. I agree to give a blood sample at the first clinic visit. INITIAL

11. **WOMEN OF CHILD BEARING POTENTIAL AT RISK OF PREGNANCY:** INITIAL
    - I agree to take a pregnancy test at the first clinic visit
    - I agree to use my usual hormonal or barrier method of contraception as detailed in the patient information sheet
    - I agree to refrain from donation of eggs during the first 6 months of the study

12. I understand that my pseudonymised data will be held on servers located within and outside of the EU, and that access to data managed by Southampton Clinical Trials Unit (SCTU) will be strictly controlled and applicable Data Protection Legislation will be abided by. INITIAL

13. **I agree to take part in the SAFA study.** INITIAL

	YES	NO
14. **<u>OPTIONAL</u>**: If I withdraw from the study, I give permission for the study team to still use my pseudonymised data that has been collected.	INITIAL	INITIAL
15. **<u>OPTIONAL</u>:** I agree that the hospital may store photographs of my facial acne taken at my first clinic visit.	INITIAL	INITIAL
16. **<u>OPTIONAL</u>:** I agree to being informed of the results of the SAFA study.	INITIAL	INITIAL

____________________	____________________	____________________
Name of Participant	Signature	Date (DD-MMM-YYYY)

____________________	____________________	____________________
Name of researcher taking consent	Signature	Date (DD-MMM-YYYY)

<u>Reminder for Research Team:</u>

- <u>Original</u> signed consent form to be kept in the <u>Investigator Site File</u>
- 1 <u>photocopy</u> given to the <u>participant</u>
- 1 <u>photocopy</u> filed in the <u>participant's medical records</u>
- 1 <u>scanned copy</u> to be <u>emailed</u> to the Southampton CTU via secure <u>nhs.net email</u> account, for central monitoring purposes

SAFA Informed Consent Form v4 05-MAR-2021.docx

Funded by NIHR
