# Supplementary material for: Spironolactone for adult female acne (SAFA): protocol for a double-blind, placebo-controlled, phase III randomised study of spironolactone as systemic therapy for acne in adult women
Source: BMJ Open. 2021 Aug 26;11(8):e053876. doi: 10.1136/bmjopen-2021-053876 (PMC8395279; doi:10.1136/bmjopen-2021-053876)
Supplement: Author's manuscript [file bmjopen-2021-053876.draft_revisions.pdf]

BMJ Open

BMJ Open is committed to open peer review. As part of this commitment we make the peer review history of every article we publish publicly available.

When an article is published we post the peer reviewers' comments and the authors' responses online. We also post the versions of the paper that were used during peer review. These are the versions that the peer review comments apply to.

The versions of the paper that follow are the versions that were submitted during the peer review process. They are not the versions of record or the final published versions. They should not be cited or distributed as the published version of this manuscript.

BMJ Open is an open access journal and the full, final, typeset and author-corrected version of record of the manuscript is available on our site with no access controls, subscription charges or pay-per-view fees (http://bmjopen.bmj.com).

If you have any questions on BMJ Open's open peer review process please email info.bmjopen@bmj.com

BMJ Open

Spironolactone for Adult Female Acne (SAFA): A double-blind, placebo-controlled, phase III randomised study of spironolactone as systemic therapy for acne in adult women: study protocol

Journal:	*BMJ Open*
Manuscript ID	bmjopen-2021-053876
Article Type:	Protocol
Date Submitted by the Author:	26-May-2021
Complete List of Authors:	Renz, Susanne; University of Southampton, Southampton Clinical Trials Unit<br>Chinnery, Fay; University of Southampton, Southampton Clinical Trials Unit<br>Stuart, Beth; University of Southampton, Faculty of Medicine, School of Primary Care, Population Sciences and Medical Education ; University of Southampton, Southampton Clinical Trials Unit<br>Day, Laura; University of Southampton, Southampton Clinical Trials Unit<br>Muller, Ingrid; University of Southampton, Faculty of Medicine, School of Primary Care, Population Sciences and Medical Education<br>Soulsby, Irene; PPI representative<br>Nuttall, Jacqui; University of Southampton, Southampton Clinical Trials Unit<br>Thomas, Karen ; PPI representative, Acne Support<br>Thomas, Kim; University of Nottingham, School of Medicine, Centre of Evidence Based Dermatology<br>Sach, Tracey; University of East Anglia, Health Economics Group, Norwich Medical School<br>Stanton, Louise; University of Southampton, Southampton Clinical Trials Unit<br>Ridd, Matthew; University of Bristol Faculty of Health Sciences, Population Health Sciences<br>Francis, Nick; University of Southampton, School of Primary Care Population Sciences and Medical Education<br>Little, Paul; University of Southampton, Faculty of Medicine, School of Primary Care, Population Sciences and Medical Education<br>Eminton, Zina; University of Southampton, Southampton Clinical Trials Unit<br>Griffiths, Gareth; University of Southampton, Southampton Clinical Trials Unit<br>Layton, Alison M; University of York, Hull York Medical School<br>Santer, Miriam; University of Southampton, Faculty of Medicine, School of Primary Care, Population Sciences and Medical Education
Keywords:	DERMATOLOGY, Acne < DERMATOLOGY, Adult dermatology < DERMATOLOGY

For peer review only - http://bmjopen.bmj.com/site/about/guidelines.xhtml

For peer review only - http://bmjopen.bmj.com/site/about/guidelines.xhtml

I, the Submitting Author has the right to grant and does grant on behalf of all authors of the Work (as defined in the below author licence), an exclusive licence and/or a non-exclusive licence for contributions from authors who are: i) UK Crown employees; ii) where BMJ has agreed a CC-BY licence shall apply, and/or iii) in accordance with the terms applicable for US Federal Government officers or employees acting as part of their official duties; on a worldwide, perpetual, irrevocable, royalty-free basis to BMJ Publishing Group Ltd ("BMJ") its licensees and where the relevant Journal is co-owned by BMJ to the co-owners of the Journal, to publish the Work in this journal and any other BMJ products and to exploit all rights, as set out in our licence.

The Submitting Author accepts and understands that any supply made under these terms is made by BMJ to the Submitting Author unless you are acting as an employee on behalf of your employer or a postgraduate student of an affiliated institution which is paying any applicable article publishing charge ("APC") for Open Access articles. Where the Submitting Author wishes to make the Work available on an Open Access basis (and intends to pay the relevant APC), the terms of reuse of such Open Access shall be governed by a Creative Commons licence – details of these licences and which Creative Commons licence will apply to this Work are set out in our licence referred to above.

Other than as permitted in any relevant BMJ Author's Self Archiving Policies, I confirm this Work has not been accepted for publication elsewhere, is not being considered for publication elsewhere and does not duplicate material already published. I confirm all authors consent to publication of this Work and authorise the granting of this licence.

For peer review only

Title

Spironolactone for Adult Female Acne (SAFA): A double-blind, placebo-controlled, phase III randomised study of spironolactone as systemic therapy for acne in adult women: study protocol

Names protocol contributors

Susanne Renz[1], Fay Chinnery[1], Beth Stuart[1,2], Laura Day[3], Ingrid Muller[2], Irene Soulsby[4], Jacqueline Nuttall[1], Karen Thomas[4], Kim S Thomas[5], Tracey Sach[6], Louise Stanton[1], Matthew J Ridd[7], Nick Francis[2], Paul Little[2], Zina Eminton[1], Gareth Griffiths[1], Alison M Layton[8], Miriam Santer[2]

Author affiliations

[1]University of Southampton, Southampton Clinical Trials Unit

[2]University of Southampton, Faculty of Medicine, School of Primary Care, Population Sciences and Medical Education

[3]Univeristy of Southampton, Southampton Clinical Trials Unit

[4]PPI contributor

[5]Centre of Evidence Based Dermatology, School of Medicine, University of Nottingham

[6]Health Economics Group, Norwich Medical School, University of East Anglia

[7]Population Health Sciences, University of Bristol

[8]Hull York Medical School, University of York

Correspondence: safa@soton.ac.uk, M.Santer@soton.ac.uk

For peer review only

For peer review only - http://bmjopen.bmj.com/site/about/guidelines.xhtml

Abstract

Introduction: Acne is one of the most common inflammatory skin diseases worldwide and can cause significant psychosocial impact and permanent physical scarring. Spironolactone, a potassium-sparing diuretic, has anti-androgenic properties, providing the potential to reduce sebum production and hyperkeratinisation in the intrafollicular duct of acne prone follicles. Dermatologists have prescribed spironolactone for acne in adult women for over 30 years, but robust clinical study data to evidence use are lacking.

Methods and analysis: Women (≥18 years) with persistent facial acne requiring systemic therapy, are randomised to receive one tablet daily of 50 mg spironolactone or a matched placebo for the first 6 weeks, increasing to up to two tablets daily (total 100 mg spironolactone or matched placebo) until week-24, along with usual topical therapy if desired. Study treatment stops at week-24, participants are informed of their treatment allocation and enter an unblinded follow-up period for up to 6 months (up to 52 weeks after baseline). Primary outcome is the Acne-specific Quality of Life (Acne-QoL) symptom subscale score at week-12. Secondary outcomes include: Acne-QoL total and subscales, participant acne self-assessment recorded on a 6-point Likert scale at 6, 12, 24 weeks and up to 52 weeks, Investigator's Global Assessment (IGA) at weeks 6 and 12, cost and cost effectiveness are assessed over 24 weeks. Aiming to detect a group difference of 2 points on the Acne-QoL symptom subscale (s.d. 5.8, effect size 0.35), allowing for 20% loss to follow-up, gives a sample size of 398 participants. **Ethics and dissemination:** This protocol was approved by Wales Research Ethics Committee (18/WA/0420). Follow-up to be completed in early 2022. Findings will be disseminated to participants, peer-reviewed journals, networks and patient groups, on social media, on the study website and the Southampton Clinical Trials Unit (CTU) website to maximise impact.

Study registration: ISRCTN 12892056.

For peer review only - http://bmjopen.bmj.com/site/about/guidelines.xhtml

Strengths and limitations of this study

- First adequately powered randomised placebo-controlled study to provide evidence about the clinical and cost-effectiveness of spironolactone in the treatment of acne
- Pragmatic design includes a primary outcome that is a participant-reported outcome measure, broad eligibility and recruitment strategies via primary care, secondary care, community and social media advertising
- Randomisation to either spironolactone or matched placebo, with participants in both groups using topical treatments as usual (creams, gels, lotions), if desired, in order to reflect the place of oral treatments in the acne care pathway
- Dosing regimen was informed by survey amongst health professionals indicating an initial dose of 50mg with an increase up to 150 mg spironolactone depending on response
- Adaptions during the COVID-19 pandemic included change to remote follow-up visits (via phone or video call), posting of study tablets/questionnaires to participants and increased social media advertising from July 2020

Keywords

Spironolactone; adult female acne; topical therapy; dermatology; randomised controlled study

Background

Acne vulgaris (from here on referred to as acne) is the eighth most common disease worldwide[1] and typically starts in adolescence with 15-20% of people affected showing moderate or severe acne, often persisting into adulthood[2]. Facial scarring occurs in approximately 20% of people but the main impact is social, with levels of psychological disability equivalent to those seen in conditions such as asthma and diabetes[3,4]. Incidence of acne in adult women is considerable and growing[5-9].

First line treatment for acne is topical treatments either alone or combination preparations, containing retinoids, benzoyl peroxide and/or antibiotics[3]. However, non-adherence to topical treatments is common, possibly because of the need to be used consistently for up to 8 weeks and adverse reactions, such as stinging or redness, are common[10]. People therefore commonly seek second-line therapies, such as oral antibiotics, co-cyprindiol or combined oral contraceptives[3]. In the UK, oral isotretinoin can be used under the

For peer review only - http://bmjopen.bmj.com/site/about/guidelines.xhtml

supervision of a dermatologist for indications including severe acne or acne that has not responded to an adequate course of a systemic antibiotics. Oral isotretinoin is highly effective, but is contraindicated for some and is teratogenic[2].

A third of people who consult with acne are prescribed long courses of oral antibiotics[3,11]. However, acne is a disease of sebogenesis and antibiotics have no effect on sebum production[12,13]. Furthermore, rising rates of antibiotic resistance mean non-antibiotic alternatives are needed[14].

Spironolactone, a potassium-sparing diuretic, is widely used in the UK for indications including hypertension[15] and has been used off-license for women with acne for ≥30 years due to its anti-androgenic properties. US Guidelines suggest a role in the management of female acne[1]. Spironolactone is not used to treat acne in men, because of its feminising side effects[16].

There is limited evidence for the effectiveness of spironolactone for treating acne and the need for evidence from randomised controlled trials (RCTs) in this patient group is acknowledged[16].

When considering spironolactone as a potential alternative systemic therapy for acne, one study reported treatment success in women who had previously failed isotretinoin[17] and a second study comparing the frequency with which participants switched drug treatment within one year of initiation, demonstrated no significant difference between those taking spironolactone and those taking oral antibiotics for their acne,[18] implying they are equally tolerated by users.

A James Lind Alliance Priority Setting Partnership, funded by the National Institute for Health Research (NIHR), identified the need to establish the best way to manage acne in women who may or may not have underlying hormonal abnormalities[19]. This informed an NIHR commissioned call (16/13 Persistent acne in adult women) for proposals to answer the research question: 'What is the effectiveness of spironolactone in the treatment of moderate-severe persistent acne in adult women?'

This study will investigate the addition of spironolactone to usual topical therapy in adult women with persistent facial acne requiring systemic therapy.

Methods

Study design and setting

The study is a phase III, multicentre, double-blind, randomised superiority study, to investigate clinical and cost-effectiveness of spironolactone in the treatment of moderate or severe persistent facial acne in adult women compared to placebo, in addition to standard topical treatment. The design is pragmatic in order to

For peer review only - http://bmjopen.bmj.com/site/about/guidelines.xhtml

have strong external validity and to inform real-world decision-making for women with acne and their health professionals. Pragmatic design includes broad eligibility and recruitment strategies, a primary outcome that is relevant to participants, low intensity follow-up and an intention-to-treat (ITT) analysis. ‘Moderate to severe acne’, in the context of this study, is defined as acne that warrants treatment with oral antibiotics, as judged by the potential participant and study clinician.

Baseline and follow-up appointments are carried out by UK hospital dermatology centres in order to facilitate blood tests at baseline and clinical assessments. Participants continue on the allocated treatment (spironolactone or placebo) in combination with their usual topical treatment (if desired) for a total duration of 24 weeks with assessments at weeks 6 and 12. Primary outcome is assessed at week-12 with the patient-reported Acne-QoL[20,21]. From week-12, participants in both groups may receive ‘usual care’ from their usual clinical team including oral treatments (oral antibiotics, hormonal treatments), and from week-24 isotretinoin, if the participant and study clinician feel the need for rescue treatment. At week-24, participants stop taking their study treatment, are informed of their treatment group allocation and enter an unblinded observational follow-up period for up to six months (up to 52 weeks after baseline). During this observational final follow-up period, participants can ask their General Practitioner (GP) to be prescribed spironolactone for their acne if they wish, or pursue other acne treatments as part of usual care. The study schema (Figure 1) illustrates the patient pathway through the study with Table 1 summarising the study procedures.

Adaptations during the COVID-19 pandemic included the option to hold follow-up visits at weeks 6 and 12 remotely (phone/video calls) and to post out the study tablets and questionnaires to participants. Participants were also given standardised instructions on how to photograph their face to submit as part of remote follow-up assessments. Baseline visits continued to be face-to-face due to the requirement of a urine pregnancy test and blood test to assess kidney function and serum potassium levels.

FIGURE 1: STUDY SCHEMA

TABLE 1: SCHEDULE OF PROCEDURES

Eligibility

Inclusion criteria

Participants should fulfil all the following criteria:

- Women aged ≥18 years
- Facial acne vulgaris, symptoms present for at least 6 months

For peer review only - http://bmjopen.bmj.com/site/about/guidelines.xhtml

For peer review only

143 • Acne of sufficient severity to warrant treatment with oral antibiotics, as judged by the study clinician. 144 Patients with an IGA≥2 are eligible to participate in the study

145 • Women of childbearing potential at risk of pregnancy must be willing to use their usual hormonal or 146 barrier method of contraception for the first 6 months of the study (whilst taking the study 147 investigational medicinal product (IMP)) and for at least 4 weeks afterwards

148 • Willing to be randomised to either study group

149 • Willing and able to give informed consent

150 • Sufficient English to carry out primary outcome Acne-QoL (which has not been validated in other 151 languages)

152 **Exclusion criteria**

153 Individuals meeting any of the following criteria will be excluded:

154 • Acne grade 0-1 using IGA (i.e. clear or almost clear)

155 • Has ever taken spironolactone

156 • Oral antibiotic treatment (lasting longer than a week) for acne within past month

157 • Oral isotretinoin treatment within past 6 months

158 • Started, stopped or changed long-term (lasting more than 2 weeks) hormonal contraception, co-159 cyprindiol or other hormonal treatment within past 3 months

160 • Planning to start, stop or change long-term (lasting more than 2 weeks) hormonal contraception, co-161 cyprindiol or other hormonal treatment within the next 3 months

162 • Pregnant/breastfeeding

163 • Intending to become pregnant in the next 6 months

164 • Contra-indicated to spironolactone:

165 ○ Currently taking potassium-sparing diuretic, ACE inhibitor, angiotensin II receptor blocker or 166 digoxin

167 ○ Hereditary problems of galactose intolerance, lactase deficiency or glucose-galactose 168 malabsorption (as the spironolactone and placebo tablets contain lactose)

169 ○ Androgen-secreting adrenal or ovarian tumour

170 ○ Cushing's syndrome

171 ○ Congenital adrenal hyperplasia

For peer review only - http://bmjopen.bmj.com/site/about/guidelines.xhtml

- Estimated Glomerular Filtration Rate (eGFR) below 60 ml/min/1.73m^2
- Serum potassium level above upper limit of reference range for the laboratory processing the sample

Intervention and control

Study participants receive one tablet daily (50 mg spironolactone or matched placebo) for the first six weeks of the study. At, or any time after the week-6 visit, the dose is escalated to two tablets daily (total 100 mg spironolactone or matched placebo) by the study clinician, providing the participant is tolerating any side effects (see box 1). Participants are instructed to take their total dose once daily in the morning to avoid diuresis later in the day/evening.

Participants may use their usual topical treatments throughout the study but adherence to topicals is not actively promoted as this may mask differences between the randomised groups. Participants are discouraged from changing their topical treatments between baseline and week-12.

INSERT BOX 1: RATIONAL FOR DOSING REGIMEN

Intervention adherence

The hospital study team assess participant adherence to treatment at each study visit using pill counts. In cases where only remote visits are possible, the participant informs the study team of the number of tablets remaining. However, no additional support or activity is undertaken to encourage daily pill taking, in keeping with the trial's pragmatic design.

Randomisation and assignment to intervention group

Following consent, participants are randomised 1:1 by clinical staff at the hospital site using an independent web-based system (TENALEA) using varying blocks of size 2 and 4, stratified by centre and by baseline severity (IGA<3 vs 3 or more). The allocation sequence was generated by a statistician using computer-generated random numbers. Participants, study staff and investigators are blind to the treatment allocation until end of the treatment period at week-24. Treatment allocation is revealed prior to week-24 only if there is a clinical need to do so.

Recruitment

Potential participants, identified in primary and secondary care, direct advertising in areas close to recruiting hospitals and social media advertising, are directed to the study website

For peer review only - http://bmjopen.bmj.com/site/about/guidelines.xhtml

(https://www.southampton.ac.uk/safa) or to contact the local study team directly.

In primary care, recruitment is supported by general practices acting as Participant Identification Centres (PIC) local to the recruiting centres identifying potential participants either opportunistically or via database search based on an acne diagnosis and relevant prescription within the past 6 months and mail-out of invitation pack. In secondary care, potential participants are identified opportunistically in out-patient clinics and through screening new referral letters.

We use targeted social media advertising to promote the study, build study awareness and interest.

Participants are free to withdraw consent from the study at any time without providing a reason. They may withdraw from study treatment but remain in follow-up; withdraw from study and follow-up, but give permission for their data to be used in analyses; or completely withdraw from the study and not permit their data to be used.

Primary and secondary outcome measures

Clinically, the effectiveness of acne treatments is usually judged at 8-12 weeks, so the primary outcome in the study is assessed at week-12 with the Acne-QoL symptom subscale score[20,21]. The Acne-QoL was developed and validated for use in a clinical study to assess the impact of therapy on quality of life among people with facial acne and the primary outcome at week-12 is the symptom subscale score of the Acne-QoL, because the Acne-QoL was intended to be presented as 4 separate subscale scores. It was not designed or validated to have a total score, however, it has published Minimum Clinically Important Difference (MCID) of 2 points for the subscales and range 0 to 30 for symptom subscale score[20-22]. Other participant-reported outcome measures in acne do not have a published MCID available and have not been found to have advantages in terms of acceptability and validity [23].

Secondary outcomes include Acne-QoL at week-24, participant self-assessed overall improvement recorded on a 6-point Likert scale with photographs taken at baseline[24], IGA[5], participant global assessment, use of acne medication and participant satisfaction with study treatment. The IGA is a 5-point scale ranging from clear to severe (0 ‘Clear’; 1 ‘Almost clear’; 2 ‘Mild’; 3 ‘Moderate’; 4 ‘Severe’)[6,25]. The Investigator’s Global Assessment (IGA)[5,24] is used to grade the participant’s acne as lesion counts are time consuming, with wide inter-assessor variation and give little additional information to global assessments. All outcome measures are shown in Table 2.

For peer review only - http://bmjopen.bmj.com/site/about/guidelines.xhtml

The safety profile of spironolactone is well established[15,16]. Consequently, we collect information about adverse reactions of special interest, both to inform the dose review decision from week-6 onwards as well as to learn more about incidence of side effects in this population. We also collect and report all serious adverse events (SAEs).

TABLE 2: SCHEDULE OF OBSERVATIONS

Pregnancy

Spironolactone is considered contraindicated in pregnancy, or a category C drug (i.e. potential benefits may warrant use in pregnant women despite potential risks)[1,15]. The main concern is around possible feminisation of the male foetus in the third trimester of pregnancy[1]. Women of childbearing potential at risk of pregnancy will be asked to use their usual hormonal or barrier method of contraception during the first 24 weeks of the study and for at least 4 weeks (approximately one menstrual cycle) afterwards. A pregnancy test will be conducted for all participants at their baseline visit and documented in their medical notes. At weeks 6 and 12, the study nurse/doctor will ask participants to confirm that they are still using contraception and have not changed contraceptive method. Participants who become pregnant will be asked to inform their local site study team as soon as possible and will not be able to continue in the study.

Sample size

Based on comparison of the Acne-QoL symptom score between groups at week-12, power 90%, alpha 0.05 and seeking a difference between groups of 2 points on the symptom subscale (s.d. 5.8, effect size 0.35), 346 participants are needed. Allowing for 20% loss to follow up gives a total 434 participants (217 per group). Following discussions with oversight committees post funding award, the sample size was recalculated. Allowing for a correlation with baseline of 0.293 and a deflation factor of $1-\rho 2$[26], gives a total sample size required of 398 participants. A difference of 2 points on the symptom subscale and a standard deviation of 5.8 (equivalent to an effect size 0.35) is in line with that reported in studies in a similar patient group and with the MCID reported for Acne-QoL[20,21].

Data collection methods

Participant data are entered into study electronic case report forms (eCRF) via a remote data collection tool (Medidata Rave) by trained hospital research personnel with specific roles on the study and regularly checked for missing or anomalous values by Southampton CTU study staff.

For peer review only - http://bmjopen.bmj.com/site/about/guidelines.xhtml

Data management

Participant data are pseudonymised by assigning each participant a participant identifier code, which is used to identify the participant during the study and for any participant-specific communication between Southampton CTU and recruiting sites.

Patient and public involvement (PPI)

This study addresses a priority area identified as important to patients and health professionals by the James Lind Alliance Priority Setting Partnership for Acne[7]. We gained feedback from a virtual acne-specific patient panel, convened through 'People in Research' (https://www.peopleinresearch.org). A patient survey was carried out with the support of the UK Dermatology Clinical Trials Network (UK DCTN) in order to inform the study design. Findings suggested that participants would find it difficult to abstain from using topical treatments for more than 12 weeks and that asking participants to take a placebo for one year would also be a barrier to recruitment.

Two public contributors with experience of acne attend all Trial Management Group (TMG) meetings to ensure that decisions around study design are informed by their perspective, study procedures are feasible for participants and study materials are readable and include all the relevant information that participants would want. Public contributors influenced the trial design and delivery, for instance by advocating use of social media advertising to improve recruitment, arguing against repeated measures in this patient group and that an upper age limit of 50 years was arbitrary and could appear discriminatory.

Statistical methods

The study will be reported in accordance with CONSORT guidelines. A detailed statistical analysis plan (SAP) will be written and reviewed prior to the study database being locked.

The modified ITT population consists of all participants who have consented and been randomised to a treatment arm and have complete data for the outcome being analysed. Analyses will be performed according to the modified ITT principle using a linear regression model. All analyses will be carried out in the modified ITT population, with the level of missing data reported, unless otherwise stated. The frequency and pattern of missing data will be examined and a multiple imputation model will be used as a sensitivity analysis if appropriate.

For the primary analyses, descriptive statistics will be obtained for the randomised groups to characterise recruited participants and assess baseline comparability. For the primary outcome, a linear regression model

For peer review only - http://bmjopen.bmj.com/site/about/guidelines.xhtml

will be used to analyse Acne-QoL symptom subscale at week-12, adjusting for baseline variables (including baseline Acne-QoL symptom subscale score, use of topical treatments, use of hormonal contraception/co-cyprindiol) and randomisation stratification variables (centre, baseline severity (IGA < 3 versus 3 or more)). A full list of covariates and model specification will be set out in the SAP. A 95% confidence interval for the least squares mean difference between arms in Acne-QoL symptom subscale at week-12 will be calculated. The same analysis methods will be used to summarise Acne-QoL symptom subscale at other time points (weeks 6, 24 and up to 52 weeks after baseline) and for the other Acne-QoL subscales (self-perception, role-emotional and role-social) and total score. IGA and participants' comparison with baseline photo at weeks 6, 12 and 24 will be dichotomised as success or failure as recommended by the US Food and Drug Administration (with success for IGA and participants global assessment defined as clear or almost clear (grade 0 or 1) and at least a two-grade improvement from baseline; this represents a clinically meaningful outcome). The dichotomised outcomes will be summarised by frequencies and percentages and compared by group using logistic regression adjusting for baseline assessment, use of hormonal contraception/co-cyprindiol), use of topical treatment and randomisation stratification factors.

Adverse reactions of special interest and SAEs will be summarised by group with frequencies and percentages and compared with Pearson's χ^2 tests. Logistic regression modelling will also be used to adjust for any important differences in topical treatment use by group. Subgroup analyses will investigate how the treatment effect differs by whether participants have symptoms consistent with polycystic ovary syndrome (PCOS) as recorded at the baseline visit. It is acknowledged the study is not powered for this subgroup analysis. The same analysis methods will be applied to the outcomes collected at up to 52 weeks however the interpretation of these results will be assessed with caution as participants will potentially have been off treatment for up to 6 months or started a different acne treatment. All analyses will be carried out using STATA and/or SAS.

There are no planned interim analyses or subgroup analyses.

Health economics analysis

If the intervention is found effective, a within-study economic evaluation will be undertaken to assess value for money of spironolactone plus usual care versus placebo plus usual care. The main perspective of the analysis will be that of the NHS over the 24 week treatment period, although a secondary analysis will assess the

For peer review only

For peer review only - http://bmjopen.bmj.com/site/about/guidelines.xhtml

importance of a broader perspective by incorporating out of pocket costs related to acne and any productivity/employment impacts for people with persistent acne[27].

Costs, including intervention and wider NHS resource use, are being recorded in the study eCRF for the former whilst wider NHS resource use is captured in participant questionnaires at baseline, weeks 6, 12, and 24. Costs will be valued using published unit costs for a common recent price year to estimate mean cost per participant in each arm[28-30].

Review of the reliability, validity and responsiveness of three generic preference-based measures (EQ-5D, SF-6D and HUI) in skin conditions only found evidence to support the use of the EQ-5D in skin diseases with no studies looking at measurement properties for the Short-Form six-dimensions (SF-6D) or Health Utilities Index (HUI) in skin disease[31]. Problems on the EQ-5D domains were found to be substantially higher in the acne sample receiving specialist care than in an age truncated population sample (aged 20-39 years) particularly on the pain/discomfort (42.1% in the acne sample versus 17.7% in an age-truncated population sample) and anxiety/depression domains (52.8% versus 12.5% respectively)[32]. EQ-5D was found to be responsive to change, with moderate effect sizes at 4 and 12 months (-0.44 and -0.53 respectively)[32]. We will value the EQ-5D-5L in our primary analysis in line with NICE recommendations at the time of analysis[3,33]. Quality Adjusted Life Years (QALYs) (estimated using EQ-5D-5L[31,32]) for the study period will be estimated using linear interpolation and area under the curve with and without baseline adjustment[34]. Clinical measures were found to be more responsive to change than the generic measures (shown by larger effect sizes) and combination of generic preference based measures with the use of disease specific measure was concluded to be desirable[32]. The primary economic evaluation will be an incremental cost utility analysis to enable the cost effectiveness to be compared across a range of health conditions and interventions such that decision makers can use the information to inform prioritisation of health care. A secondary cost effectiveness analysis using the disease specific Acne-QoL will be presented as appropriate, though it should be noted that this instrument does not have utility weights available and it is unclear what incremental cost per unit of change on the Acne-QoL represents good value for money. All analysis will be conducted and presented using established methods[27,35].

If spironolactone is not found to be clinically effective a full economic evaluation will not be conducted. Instead, estimates of mean costs and utility per participant will be presented at the various study time points as these may be informative for other researchers undertaking future economic studies or economic modelling in this clinical area.

For peer review only - http://bmjopen.bmj.com/site/about/guidelines.xhtml

A detailed Health Economic Analysis Plan will be written prior to the study database being locked.

Oversight and monitoring

The TMG includes representatives with expertise in dermatology, primary care research, psychology, medical statistics and health economics, public contributors, and Southampton CTU staff involved in the day-to-day running of the study and is responsible for the oversight of the progress of the study. An independent Trial Steering Committee (TSC) and an independent Data Monitoring and Ethics Committee (DMEC) have been set up to monitor study progress and safety.

Data on adverse reactions will be collected at the baseline and follow-up visits, and participants will be asked to report any adverse reactions in their week-24 questionnaire. SAEs may be identified by participant report at any time directly to the hospital study team, at follow-up visits or questionnaires. Participants' GPs will be informed of their patient's participation in the study and asked to notify the hospital study team of any potential SAE. The study also has a UK regulatory compliant real-time SAE reporting process to identify serious adverse reactions and suspected unexpected serious adverse reactions that could suspend or stop the study if warranted.

The Southampton CTU has undertaken a risk assessment for the study which includes the requirements for monitoring (both central and site). The Southampton CTU undertakes a number of internal audits of its own systems and processes annually and has routine audits from both its sponsor and the independent Medicine and Health care products regulatory authority (MHRA) every 2-3 years.

Discussion

This is the first adequately powered pragmatic, randomised trial investigating the effect of spironolactone on acne in adult women in comparison to a matched placebo. If found to be clinically and cost effective, use of spironolactone will likely become the new standard of care in addition to topical treatments potentially reducing antibiotic use for women requiring systemic therapy.

Respondents to a survey of people with experience with acne reported that they would be unwilling to be recruited to a study where they remained blinded to the treatment allocation for 52 weeks, due to concerns about potential worsening of acne over this time. Therefore, the study was designed as blinded treatment phase of 24 weeks with an observational follow-up period for up to 6 months after. Use of acne treatments, such as oral isotretinoin or antibiotics, between week-24 and up to 52 weeks are carefully recorded as

For peer review only

For peer review only - http://bmjopen.bmj.com/site/about/guidelines.xhtml

differences between groups would be important in interpreting week-52 outcomes. Use of topical treatments is allowed in both groups during the 24 week treatment phase as i) women with moderate-severe acne may be unwilling to be randomised to placebo alone, and ii) recruiting women with moderate-severe acne to a placebo-controlled study with no effective treatment for 12 weeks in the control arm may risk worsening of acne and possible scarring.

Although others are seeking to evaluate the role of spironolactone in comparison with oral tetracyclines[36], this is the largest study to date to inform clinical practice over the effectiveness of spironolactone as an alternative treatment for acne in adult women.

Abbreviations

ACE	Angiotensin-converting enzyme
CRN	Clinical Research Network
CTU	Clinical Trials Unit
DMEC	Data Monitoring and Ethics Committee
eCRF	Electronic Case Report Form
eGFR	Estimated Glomerular Filtration Rate
EQ-5D-5 L	EuroQol Five Dimensions Five Level
GP	General Practitioner
HUI	Health Utilities Index
HRA	Health Research Authority
HTA	Health Technology Assessment
ICF	Informed Consent Form
IGA	Investigator's Global Assessment
IMP	Investigational Medicinal Product
IPD	Individual Patient Data

For peer review only - http://bmjopen.bmj.com/site/about/guidelines.xhtml

ITT	Intention-to-treat
MCID	Minimum Clinically Important Difference
MHRA	Medicines and Healthcare products Regulatory Agency
NHS	National Health Service
NICE	National Institute for Health and Care Excellence
NIHR	National Institute for Health Research
PCOS	Polycystic Ovary Syndrome
PGA	Participant's Global Assessment
PIC	Participant Identification Centre
PIS	Participant Information Sheet
PPI	Patient and Public Involvement
PSS	Personal Social Services
QALYs	Quality Adjusted Life Years
QoL	Quality of Life
RCT	Randomised Controlled Trial
REC	Research Ethics Committee
SAE	Serious Adverse Event
SF-6D	Short Form Six Dimensions
SpR	Specialty registrar
TMG	Trial Management Group
TSC	Trial Steering Committee
UHS	University Hospital Southampton NHS Foundation Trust

For peer review only

For peer review only - http://bmjopen.bmj.com/site/about/guidelines.xhtml

UK DCTN UK Dermatology Clinical Trials Network

Declarations

Acknowledgements

- The virtual patient panel (brought together by NIHR INVOLVE) for their advice on the study design.
- The study was developed with support from the UK Dermatology Clinical Trials Network (UK DCTN). The UK DCTN is grateful to the British Association of Dermatologists and the University of Nottingham for financial support of the Network. UK DCTN conducted surveys among patients with acne and health professionals managing acne to inform the study design, promoting the study and identifying and advising on potential hospital sites delivering the study.
- Wessex CRN for funding the initial social media advertising campaign.
- Jessica Boxall and Liz Allaway for management of the study social media accounts as well as running and coordinating the social media adverts.
- Hospital dermatology centres recruiting for the study: Queen Elizabeth Hospital, Birmingham; Bristol Royal Infirmary Dermatology Centre, Bristol; University Hospital of Wales, Cardiff; General Hospital, Epsom; District Hospital, Harrogate; St Mary's Hospital (Imperial College NHS Healthcare Trust), London; Queen's Medical Centre, Nottingham; General Hospital, Poole; St Mary's General Hospital Dermatology Centre, Portsmouth; Swansea Bay University Health Board, Swansea.
- Participant Identification Centres (PICs) for searching their patient lists and mail outs and Clinical Research Networks (CRNs) for helping to identify potential PICs.

Authors' contributions

Research funding was obtained by MS, AL, NF, GG, PL, IM, JN, MJR, TS, IS, LS, BS, KT and KST. All authors have contributed to the development of the protocol and to the management of the study. SR leads on the day-to-day management of the study, overseen by ZE, MS, JN and BS. This paper was drafted by FC and SR with contributions from MS and all authors. All authors read and approved the final manuscript.

Funding

This project is funded by the National Institute for Health Research (NIHR) Health Technology Assessment

For peer review only

For peer review only - http://bmjopen.bmj.com/site/about/guidelines.xhtml

(HTA) programme (Grant Reference Number: 16/13/02) and supported by NIHR CTU support funding at Southampton CTU. The views expressed are those of the author(s) and not necessarily those of the NIHR or the Department of Health and Social Care. The NIHR HTA funder will play no role in the execution, analysis, interpretation of data, or study publication. The study is registered on the UK NIHR study portfolio meaning there are research nurses based at UK hospitals who help in screening potential patients to identify those eligible for the study.

Southampton CTU, a NIHR CTU support funded UK Clinical Research Collaboration registered CTU, is coordinating the study. University of Southampton is the sponsor for the study.

Trial status

This clinical trial was registered on 17-Sep-2018 (EudraCT Number: 2018-003630-33) and on 15-Oct-2018 (ISRCTN, registry number: ISRCTN 12892056). The first participant was recruited on 05-Jun-2019 and recruitment is expected to be completed on 31-Aug-2021. The current protocol is version 10, dated 08-Mar-2021. The full protocol v10 08-MAR-2021 is available as supplementary material and on request via safa@soton.ac.uk or on the study website https://www.southampton.ac.uk/safa/about/researchers-and-clinicians.page. REC/MHRA approved protocol amendments will be communicated to sites via email and updated trial documentation provided centrally via the trial website. Trial registries will be amended where relevant with explanations for these changes. End of study is defined as the date when the last point of data is collected for the last participant from their Final follow-up questionnaire.

Ethics approval

The trial received favourable ethical opinion from Wales Research Ethics Committee (18/WA/0420) and has Health Research Authority (HRA) approval (IRAS 246637).

Availability of data and materials

Data Management Plan

Full details of the data management strategy for the study are available in the SAFA data management plan, available on request via safa@soton.ac.uk.

Underlying data

Pseudonymised individual participant data (IPD) within the clinical study dataset will be available for sharing

For peer review only - http://bmjopen.bmj.com/site/about/guidelines.xhtml

For peer review only

via controlled access by authorised Southampton CTU staff (as delegated to Southampton CTU by the study sponsor). Data access can be requested via a Southampton CTU Data Release application form (available from https://www.southampton.ac.uk/ctu/about/index.page) after the trial is published. Please email the completed form to the Southampton CTU Data Release Committee Coordinator at ctu@soton.ac.uk.

Data access requests are reviewed against specific eligibility criteria by the Southampton CTU data custodian and key members of the study team, including a statistician and chief investigator or by an external Independent Review Panel. Decisions about requests are made promptly and usually no more than three months after receipt of request. Responses to all data requests, with a clear rationale for any refusals, will be sent promptly to the data requester.

Extended data

Written informed consent is obtained from participants during the baseline visit by qualified site trial team members. A copy of the study's Informed Consent Form (v4 05-MAR-2021) is available as supplementary material or on request via safa@soton.ac.uk .

Consent for publication

Responsibility for publication has been delegated to Prof Miriam Santer and Prof Alison Layton (co-Chief Investigators) and Prof Gareth Griffiths (Director of Southampton CTU), who have consented to this publication.

Competing interests

The authors have no competing interests to declare.

Authors' information (optional)

Not applicable.

Open Access

This is an Open Access article distributed in accordance with the terms of the Creative Commons Attribution (CC BY 4.0) license, which permits others to distribute, remix, adapt and build upon this work, for commercial use, provided the original work is properly cited. See: http://creativecommons.org/licenses/by/4.0/

For peer review only

For peer review only - http://bmjopen.bmj.com/site/about/guidelines.xhtml

Tables

Table 1: Schedule of procedures

**Observation/procedure**	**Person undertaking the specified event**	**Visit**				
		Screening/ Baseline	*Week 6*	*Week 12*	*End of Treatment/ Week 24 contact*	*End of Study/ Final Follow-up questionnaire (52 weeks or sooner)*[3]
**ENROLMENT**						
Informed Consent	Nurse/ Other clinician[1]	X				
Eligibility evaluation	Other clinician	X				
Participant characteristics	Nurse/Other clinician	X				
Blood pressure	Nurse/Other clinician	X				
Blood tests (serum potassium, eGFR)	Nurse/Other clinician	X				
Pregnancy test	Nurse/Other clinician	X				
**RANDOMISATION**	Nurse/Other clinician	X				
**ASSESSMENTS**						
Investigator's Global Assessment (IGA)	Nurse/ Other clinician	X	X	X		
Medical history	Participant	X				
Self-assessment - Participants Global Assessment (adapted IGA)	Participant	X	X	X	X	X
Acne Medication Use	Nurse/Other clinician/Participant	X	X	X	X	X
Other Medication Use	Nurse/Other clinician/Participant	X				
Acne-QoL	Participant	X	X	X	X	X
EQ-5D-5L	Participant	X	X	X	X	X
Resource use questionnaire	Participant	X	X	X	X	X
Self-assessment – comparison with baseline photo - 6 Point Likert Scale	Participant		X	X	X	X
Collection of ARs of special interest:<br>Headache<br>Dizziness/vertigo/<br>Light headedness<br>Tingling<br>Indigestion/heartburn/<br>Dyspepsia<br>Diarrhoea<br>Polyuria (passing much more urine than normal)<br>Nausea/feeling sick<br>Vomiting/being sick<br>Tenderness of the breasts<br>Breast enlargement<br>Irregular menstrual periods<br>Abdominal pain<br>Weight gain<br>Reduced libido (reduced interest in sex)<br>Fatigue/tiredness<br>Drowsiness/sleepiness	Participant/ Other clinician		X	X	X	

For peer review only

For peer review only - http://bmjopen.bmj.com/site/about/guidelines.xhtml

Observation/procedure	Person undertaking the specified event	Visit				
		Screening/ Baseline	*Week 6*	*Week 12*	*End of Treatment/ Week 24 contact*	*End of Study/ Final Follow-up questionnaire (52 weeks or sooner)*[3]
Serious Adverse Events	Other clinician (PI or delegate)		X	X	X	
Assessment of treatment response to determine dose adjustment[2]	Participant / Other clinician		X	X		
Satisfaction with study treatment	Participant				X	
**OTHER ACTIVITIES**						
Discuss use of contraception	Nurse/Other clinician	X	X	X		
Photographs of face taken	Nurse/Other clinician	X				
Photographs given to participant	Nurse/Other Clinician	X		X (If a set was stored at site)		
Letter to participant's GP (patient participation)	Nurse/Other clinician	X				
Check participant is not using oral acne treatment	Nurse/Other clinician/Participant		X	X		
Return excess IMP to clinic	Participant		X	X	X (return via post)	
Spironolactone/Placebo pill count	Nurse/Other clinician/Participant		X	X	X	
Letter to participant's GP (if dose is change)	Nurse/Other clinician		X	X		
Reminder to participant to report any subsequent adverse event(s) that might reasonably be related to participation in this study (up to 52 weeks)	Nurse/Other clinician			X		
Ask participant if they would like to receive a summary of the study results, when available	Nurse/Other clinician			X		
Letter to participant (unblinding)					X (after 24 weeks)	
Letter to participant's GP (unblinding)					X (after 24 weeks)	

[1] Dermatologist or Clinical Research Fellow, in line with local procedures with demonstrable and appropriate level of training. Specific duties delegated by the PI and listed on the delegation log.
[2]Dose escalated to 2 tablets per day if participant is tolerating side effects.
[3] The follow-up questionnaire will be sent out 6 months or sooner after the 24-week timepoint.

For peer review only - http://bmjopen.bmj.com/site/about/guidelines.xhtml

Table 2: Schedule of observations

Outcome measure	6 weeks	12 weeks (primary endpoint)	24 weeks (end of treatment)	Unblinded Follow-up (52 weeks or sooner)[h]
**Primary outcome measure**				
Acne-QoL symptom subscale score		X		
**Secondary outcome measures**				
Acne-QoL symptom subscale score	X	X	X	X
Acne-QoL other subscales[a]	X	X	X	X
Acne-QoL total score	X	X	X	X
Participant self-assessed overall improvement[b]	X	X	X	X
Investigator's Global Assessment (IGA)[c]	X	X		
Participant's Global Assessment (PGA)[d]	X	X	X	X
Participant satisfaction with study treatment[e]			X	
Health-related quality of life using EQ-5D-5L[f]	X	X	X	X
Costs incurred	X	X	X	X
Cost-effectiveness[g]			X	

[a] Self-perception, role-emotional and role-social; [b] recorded on a 6-point Likert scale with photographs taken at the baseline visit to aid recall[37]; [c] 5-point scale ranging from clear to severe (0 - Clear; 1 - Almost clear; 2 - Mild; 3 - Moderate; 4 - Severe)[38]; [d] Same scale as the IGA, but written in Plain English for participants' use; [e] Asked prior to revealing treatment allocation after 24 weeks; [f] The EQ-5D-5 L assesses five dimensions: mobility, self-care, usual activities, pain/discomfort and anxiety/depression; [g] Using EQ-5D-5L and data on health resource use during the study; [h] The follow-up questionnaire will be sent out 6 months or sooner after the 24-week timepoint.

For peer review only - http://bmjopen.bmj.com/site/about/guidelines.xhtml

BOX 1

Rationale for the dosing regimen

We conducted a survey of health professionals to inform the spironolactone dose regimen (unpublished). Responses were received from 41 Dermatology consultants, 10 Dermatology nurses and 3 Dermatology SpRs.

Of these 54 Dermatology health professionals, 22 prescribed spironolactone (9 rarely, 10 sometimes and 3 often). Most of those who responded stated that they would start at 50mg and increase up to 100-150mg depending on response. Several noted that this would depend on the patient's weight, with the starting dose lowered to 25mg if needed and allowing the dosage to increase up to 200mg. There was no consistency on the timeframe for these increases with 4 weeks, 6 weeks, 12 weeks and 6 months all being mentioned as review points.

A previous HTA study examining common treatments in the management of acne suggested that assessing efficacy at 6 weeks was ideal[24] – this informed the timing of follow-up assessments and dose escalation. US guidelines note that studies have been carried out using spironolactone doses ranging from 50mg to 200mg daily.[1] No specific dose is recommended but it is noted that side effects are dose-related.[1]

A recent hybrid systematic review of RCTs and case series identified some very low-quality evidence which showed that a daily dose of 200mg was statistically significantly more effective than placebo versus inflamed lesions, but it also confirmed that this dose is associated with a significantly greater risk of adverse side effects than lower doses.[17]

Hence, there would appear to be no merit in using these higher doses for managing acne. Data from the multiple case series suggested that any future RCT examining lower doses is likely to generate results that confirm the effectiveness and better safety profile of doses ≤100 mg per day, which informed the dosage regimen.

For most licensed indications for spironolactone, the British National Formulary states a starting dose of 100mg, titrated as required. Therefore, a starting does of 50mg in the SAFA study seems conservative.

For peer review only - http://bmjopen.bmj.com/site/about/guidelines.xhtml

References

1. Zaenglein AL, Pathy AL, Schlosser BJ, et al. Guidelines of care for the management of acne vulgaris. *J Am Acad Dermatol* 2016;74(5):945-73.e33.
2. Williams HC, Dellavalle RP, Garner S. Acne vulgaris. *The Lancet* 2012;379(9813):361-72.
3. NICE. Clinical Knowledge Summaries: Acne vulgaris. (accessed 01 July 2019).
4. Tan J, Kang S, Leyden J. Prevalence and Risk Factors of Acne Scarring Among Patients Consulting Dermatologists in the USA. *J Drugs Dermatol* 2017;16(2):97-102.
5. Holzmann R, Shakery K. Postadolescent acne in females. *Skin Pharmacol Physiol* 2014;27 Suppl 1:3-8.
6. Kim GK, Michaels BB. Post-adolescent acne in women: more common and more clinical considerations. *J Drugs Dermatol* 2012;11(6):708-13.
7. Perkins AC, Maglione J, Hillebrand GG, et al. Acne vulgaris in women: prevalence across the life span. *J Womens Health (Larchmt)* 2012;21(2):223-30.
8. Tan JK, Li Y, Fung K, et al. Divergence of demographic factors associated with clinical severity compared with quality of life impact in acne. *J Cutan Med Surg* 2008;12(5):235-42.
9. Lynn DD, Umari T, Dunnick CA, et al. The epidemiology of acne vulgaris in late adolescence. *Adolesc Health Med Ther* 2016;7:13-25.
10. Thiboutot D, Dréno B, Layton A. Acne counseling to improve adherence. *Cutis* 2008;81(1):81-6.
11. Francis NA, Entwistle K, Santer M, et al. The Management of Acne Vulgaris in Primary Care: A cohort study of consulting and prescribing patterns using CPRD. *British Journal of Dermatology* 2016:n/a-n/a.
12. Del Rosso JQ, Webster GF, Rosen T, et al. Status Report from the Scientific Panel on Antibiotic Use in Dermatology of the American Acne and Rosacea Society: Part 1: Antibiotic Prescribing Patterns, Sources of Antibiotic Exposure, Antibiotic Consumption and Emergence of Antibiotic Resistance, Impact of Alterations in Antibiotic Prescribing, and Clinical Sequelae of Antibiotic Use. *J Clin Aesthet Dermatol* 2016;9(4):18-24.
13. Khondker L, Khan SI. Acne vulgaris related to androgens - a review. *Mymensingh Med J* 2014;23(1):181-5.
14. Walsh TR, Efthimiou J, Dréno B. Systematic review of antibiotic resistance in acne: an increasing topical and oral threat. *Lancet Infect Dis* 2016;16(3):e23-33.
15. Formulary BN. *British Medical Association and the Royal Pharmaceutical Society*. http://www.bnf.org.
16. Layton AM, Eady EA, Whitehouse H, et al. Oral Spironolactone for Acne Vulgaris in Adult Females: A Hybrid Systematic Review. *American journal of clinical dermatology* 2017;18(2):169-91.
17. Isvy-Joubert A, Nguyen JM, Gaultier A, et al. Adult female acne treated with spironolactone: a retrospective data review of 70 cases. *Eur J Dermatol* 2017;27(4):393-98.
18. Barbieri JS, Choi JK, Mitra N, et al. Frequency of Treatment Switching for Spironolactone Compared to Oral Tetracycline-Class Antibiotics for Women With Acne: A Retrospective Cohort Study 2010-2016. *J Drugs Dermatol* 2018;17(6):632-38.
19. Layton A, Eady EA, Peat M, et al. Identifying acne treatment uncertainties via a James Lind Alliance Priority Setting Partnership. *BMJ Open* 2015;5(7):e008085.
20. Fehnel SE, McLeod LD, Brandman J, et al. Responsiveness of the Acne-Specific Quality of Life Questionnaire (Acne-QoL) to treatment for acne vulgaris in placebo-controlled clinical trials. *Qual Life Res* 2002;11(8):809-16.
21. McLeod LD, Fehnel SE, Brandman J, et al. Evaluating minimal clinically important differences for the acne-specific quality of life questionnaire. *PharmacoEconomics* 2003;21(15):1069-79.
22. Martin AR, Lookingbill DP, Botek A, et al. Health-related quality of life among patients with facial acne -- assessment of a new acne-specific questionnaire. *Clin Exp Dermatol* 2001;26(5):380-5.
23. Hornsey S, Stuart B, Muller I, et al. Patient-reported outcome measures for acne: a mixed-methods validation study (acne PROMs). *BMJ Open* 2021;11(3):e034047.
24. Ozolins M, Eady EA, Avery A, et al. Randomised controlled multiple treatment comparison to provide a cost-effectiveness rationale for the selection of antimicrobial therapy in acne. *Health Technol Assess* 2005;9(1):iii-212.

For peer review only - http://bmjopen.bmj.com/site/about/guidelines.xhtml

25. Tan JK, Tang J, Fung K, et al. Development and validation of a comprehensive acne severity scale. *J Cutan Med Surg* 2007;11(6):211-6.
26. Borm GF, Fransen J, Lemmens WA. A simple sample size formula for analysis of covariance in randomized clinical trials. *J Clin Epidemiol* 2007;60(12):1234-8.
27. Ramsey SD, Willke RJ, Glick H, et al. Cost-effectiveness analysis alongside clinical trials II-An ISPOR Good Research Practices Task Force report. *Value Health* 2015;18(2):161-72.
28. Curtis L, Burns A. *Unit Costs of Health and Social Care*. http://www.pssru.ac.uk/project-pages/unit-costs/2016/
29. Department of H. NHS reference costs 2015-16. https://www.gov.uk/government/publications/nhs-reference-costs-2015-to-2016. *Department of Health* Accessed 23 November 2017.
30. Health and Social Care Information C. Prescription Cost Analysis. http://data.gov.uk/dataset/prescription-cost-analysis-england. Accessed 23 November 2017.
31. Yang Y, Brazier J, Longworth L. EQ-5D in skin conditions: an assessment of validity and responsiveness. *Eur J Health Econ* 2015;16(9):927-39.
32. Klassen AF, Newton JN, Mallon E. Measuring quality of life in people referred for specialist care of acne: comparing generic and disease-specific measures. *J Am Acad Dermatol* 2000;43(2 Pt 1):229-33.
33. Devlin NJ, Shah KK, Feng Y, et al. Valuing health-related quality of life: An EQ-5D-5L value set for England. *Health Economics* 2018;27(1):7-22.
34. Manca A, Hawkins N, Sculpher MJ. Estimating mean QALYs in trial-based cost-effectiveness analysis: the importance of controlling for baseline utility. *Health Econ* 2005;14(5):487-96.
35. Drummond MF, Sculpger MJ, Claxton K, et al. *Methods for the economic evaluation of health care programmes.* : Oxford University Press; 2015.
36. Poinas A, Lemoigne M, Le Naour S, et al. FASCE, the benefit of spironolactone for treating acne in women: study protocol for a randomized double-blind trial. *Trials* 2020;21(1):571.

For peer review only

For peer review only - http://bmjopen.bmj.com/site/about/guidelines.xhtml

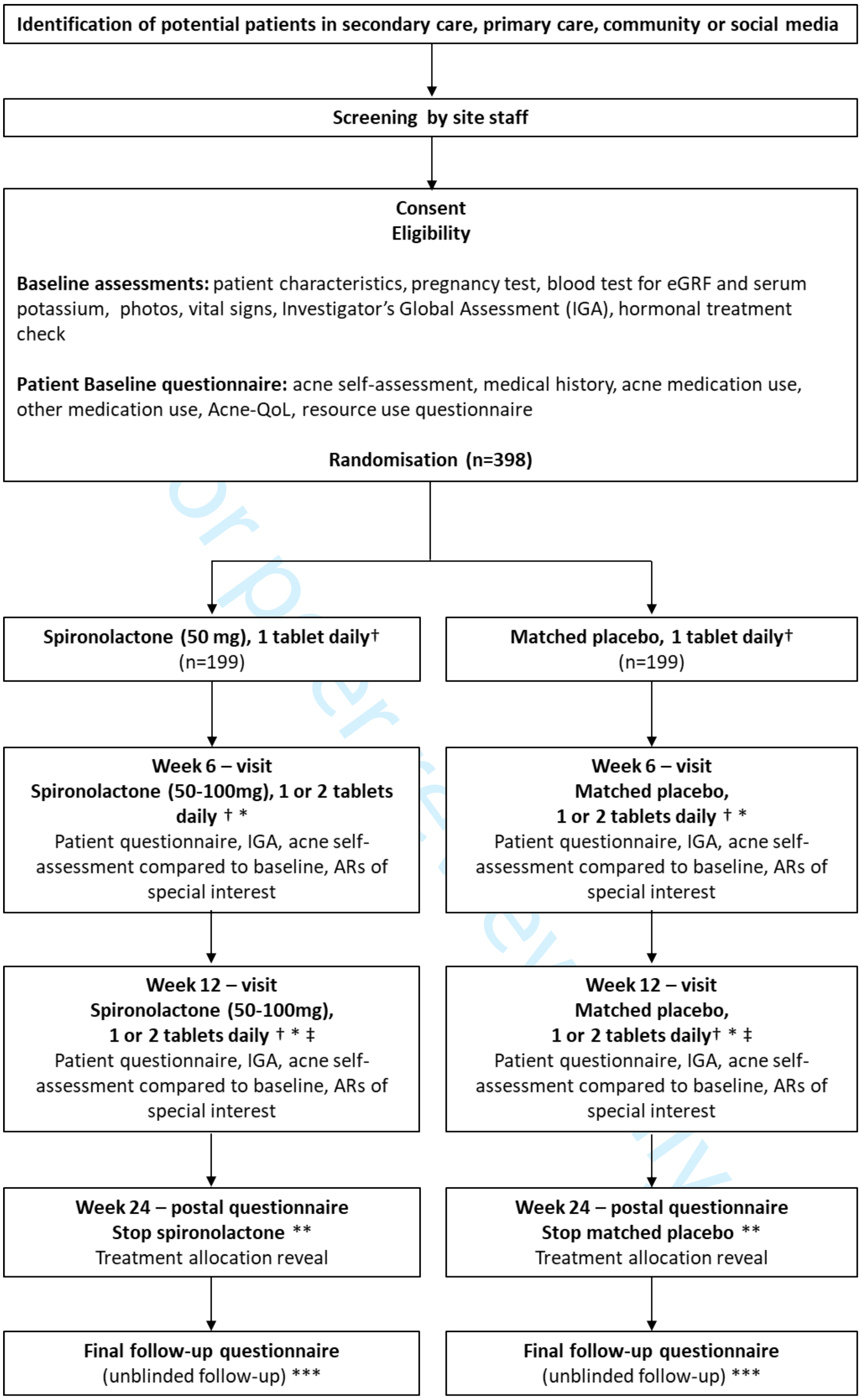

† Allow use of topical therapy (creams/lotions/gels)
*Escalate dose if study tablet is tolerated, otherwise maintain on 1 tablet
‡ Add antibiotic taken by mouth/change topical therapy if response to study tablet is inadequate
**Participants in either arm may seek to use spironolactone after this time.
***The follow-up questionnaire will be sent out 6 months or sooner after the 24-week timepoint.

For peer review only - http://bmjopen.bmj.com/site/about/guidelines.xhtml

SPIRIT

STANDARD PROTOCOL ITEMS: RECOMMENDATIONS FOR INTERVENTIONAL TRIALS

SPIRIT 2013 Checklist: Recommended items to address in a clinical trial protocol and related documents*

Section/item	ItemNo	Description	Adressed on page number
**Administrative information**			
Title	1	Descriptive title identifying the study design, population, interventions, and, if applicable, trial acronym	1
Trial registration	2a	Trial identifier and registry name. If not yet registered, name of intended registry	2, 17
	2b	All items from the World Health Organization Trial Registration Data Set	Included throughout manuscript
Protocol version	3	Date and version identifier	17
Funding	4	Sources and types of financial, material, and other support	17
Roles and responsibilities	5a	Names, affiliations, and roles of protocol contributors	1, 16
	5b	Name and contact information for the trial sponsor	17
	5c	Role of study sponsor and funders, if any, in study design; collection, management, analysis, and interpretation of data; writing of the report; and the decision to submit the report for publication, including whether they will have ultimate authority over any of these activities	17
	5d	Composition, roles, and responsibilities of the coordinating centre, steering committee, endpoint adjudication committee, data management team, and other individuals or groups overseeing the trial, if applicable (see Item 21a for data monitoring committee)	13
**Introduction**			

For peer review only

Background and rationale	6a	Description of research question and justification for undertaking the trial, including summary of relevant studies (published and unpublished) examining benefits and harms for each intervention	3-4
	6b	Explanation for choice of comparators	5
Objectives	7	Specific objectives or hypotheses	4
Trial design	8	Description of trial design including type of trial (eg, parallel group, crossover, factorial, single group), allocation ratio, and framework (eg, superiority, equivalence, noninferiority, exploratory)	5
**Methods: Participants, interventions, and outcomes**			
Study setting	9	Description of study settings (eg, community clinic, academic hospital) and list of countries where data will be collected. Reference to where list of study sites can be obtained	4-5
Eligibility criteria	10	Inclusion and exclusion criteria for participants. If applicable, eligibility criteria for study centres and individuals who will perform the interventions (eg, surgeons, psychotherapists)	5-7
Interventions	11a	Interventions for each group with sufficient detail to allow replication, including how and when they will be administered	7
	11b	Criteria for discontinuing or modifying allocated interventions for a given trial participant (eg, drug dose change in response to harms, participant request, or improving/worsening disease)	9, 5
	11c	Strategies to improve adherence to intervention protocols, and any procedures for monitoring adherence (eg, drug tablet return, laboratory tests)	7
	11d	Relevant concomitant care and interventions that are permitted or prohibited during the trial	5
Outcomes	12	Primary, secondary, and other outcomes, including the specific measurement variable (eg, systolic blood pressure), analysis metric (eg, change from baseline, final value, time to event), method of aggregation (eg, median, proportion), and time point for each outcome. Explanation of the clinical relevance of chosen efficacy and harm outcomes is strongly recommended	8, 9

For peer review only - http://bmjopen.bmj.com/site/about/guidelines.xhtml

Participant timeline	13	Time schedule of enrolment, interventions (including any run-ins and washouts), assessments, and visits for participants. A schematic diagram is highly recommended (see Figure)	7, Figure 1, Table 2
Sample size	14	Estimated number of participants needed to achieve study objectives and how it was determined, including clinical and statistical assumptions supporting any sample size calculations	9
Recruitment	15	Strategies for achieving adequate participant enrolment to reach target sample size	7, 8
**Methods: Assignment of interventions (for controlled trials)**			
Allocation:			
Sequence generation	16a	Method of generating the allocation sequence (eg, computer-generated random numbers), and list of any factors for stratification. To reduce predictability of a random sequence, details of any planned restriction (eg, blocking) should be provided in a separate document that is unavailable to those who enrol participants or assign interventions	9
Allocation concealment mechanism	16b	Mechanism of implementing the allocation sequence (eg, central telephone; sequentially numbered, opaque, sealed envelopes), describing any steps to conceal the sequence until interventions are assigned	9
Implementation	16c	Who will generate the allocation sequence, who will enrol participants, and who will assign participants to interventions	9
Blinding (masking)	17a	Who will be blinded after assignment to interventions (eg, trial participants, care providers, outcome assessors, data analysts), and how	9
	17b	If blinded, circumstances under which unblinding is permissible, and procedure for revealing a participant's allocated intervention during the trial	9
**Methods: Data collection, management, and analysis**			

Data collection methods	18a	Plans for assessment and collection of outcome, baseline, and other trial data, including any related processes to promote data quality (eg, duplicate measurements, training of assessors) and a description of study instruments (eg, questionnaires, laboratory tests) along with their reliability and validity, if known. Reference to where data collection forms can be found, if not in the protocol	9
	18b	Plans to promote participant retention and complete follow-up, including list of any outcome data to be collected for participants who discontinue or deviate from intervention protocols	4, 7
Data management	19	Plans for data entry, coding, security, and storage, including any related processes to promote data quality (eg, double data entry; range checks for data values). Reference to where details of data management procedures can be found, if not in the protocol	10
Statistical methods	20a	Statistical methods for analysing primary and secondary outcomes. Reference to where other details of the statistical analysis plan can be found, if not in the protocol	10, 11
	20b	Methods for any additional analyses (eg, subgroup and adjusted analyses)	11, 12
	20c	Definition of analysis population relating to protocol non-adherence (eg, as randomised analysis), and any statistical methods to handle missing data (eg, multiple imputation)	10, 11
**Methods: Monitoring**			
Data monitoring	21a	Composition of data monitoring committee (DMC); summary of its role and reporting structure; statement of whether it is independent from the sponsor and competing interests; and reference to where further details about its charter can be found, if not in the protocol. Alternatively, an explanation of why a DMC is not needed	13
	21b	Description of any interim analyses and stopping guidelines, including who will have access to these interim results and make the final decision to terminate the trial	10, 11

For peer review only - http://bmjopen.bmj.com/site/about/guidelines.xhtml

Harms	22	Plans for collecting, assessing, reporting, and managing solicited and spontaneously reported adverse events and other unintended effects of trial interventions or trial conduct	13
Auditing	23	Frequency and procedures for auditing trial conduct, if any, and whether the process will be independent from investigators and the sponsor	13
**Ethics and dissemination**			
Research ethics approval	24	Plans for seeking research ethics committee/institutional review board (REC/IRB) approval	2, 17
Protocol amendments	25	Plans for communicating important protocol modifications (eg, changes to eligibility criteria, outcomes, analyses) to relevant parties (eg, investigators, REC/IRBs, trial participants, trial registries, journals, regulators)	17
Consent or assent	26a	Who will obtain informed consent or assent from potential trial participants or authorised surrogates, and how (see Item 32)	18, Table 1
	26b	Additional consent provisions for collection and use of participant data and biological specimens in ancillary studies, if applicable	n/a
Confidentiality	27	How personal information about potential and enrolled participants will be collected, shared, and maintained in order to protect confidentiality before, during, and after the trial	17, 18
Declaration of interests	28	Financial and other competing interests for principal investigators for the overall trial and each study site	18
Access to data	29	Statement of who will have access to the final trial dataset, and disclosure of contractual agreements that limit such access for investigators	18
Ancillary and post-trial care	30	Provisions, if any, for ancillary and post-trial care, and for compensation to those who suffer harm from trial participation	n/a

For peer review only

Dissemination policy	31a	Plans for investigators and sponsor to communicate trial results to participants, healthcare professionals, the public, and other relevant groups (eg, via publication, reporting in results databases, or other data sharing arrangements), including any publication restrictions	2
	31b	Authorship eligibility guidelines and any intended use of professional writers	16
	31c	Plans, if any, for granting public access to the full protocol, participant-level dataset, and statistical code	17
**Appendices**			
Informed consent materials	32	Model consent form and other related documentation given to participants and authorised surrogates	Additional File 2
Biological specimens	33	Plans for collection, laboratory evaluation, and storage of biological specimens for genetic or molecular analysis in the current trial and for future use in ancillary studies, if applicable	n/a

*It is strongly recommended that this checklist be read in conjunction with the SPIRIT 2013 Explanation & Elaboration for important clarification on the items. Amendments to the protocol should be tracked and dated. The SPIRIT checklist is copyrighted by the SPIRIT Group under the Creative Commons "Attribution-NonCommercial-NoDerivs 3.0 Unported" license.

For peer review only

BMJ Open

Spironolactone for Adult Female Acne (SAFA): A double-blind, placebo-controlled, phase III randomised study of spironolactone as systemic therapy for acne in adult women: study protocol

Journal:	*BMJ Open*
Manuscript ID	bmjopen-2021-053876.R1
Article Type:	Protocol
Date Submitted by the Author:	14-Jul-2021
Complete List of Authors:	Renz, Susanne; University of Southampton, Southampton Clinical Trials Unit<br>Chinnery, Fay; University of Southampton, Southampton Clinical Trials Unit<br>Stuart, Beth; University of Southampton, Primary Care Research Centre, Faculty of Medicine, School of Primary Care, Population Sciences and Medical Education ; University of Southampton, Southampton Clinical Trials Unit<br>Day, Laura; University of Southampton, Southampton Clinical Trials Unit<br>Muller, Ingrid; University of Southampton, Primary Care Research Centre, Faculty of Medicine, School of Primary Care, Population Sciences and Medical Education<br>Soulsby, Irene; PPI representative<br>Nuttall, Jacqui; University of Southampton, Southampton Clinical Trials Unit<br>Thomas, Karen ; PPI representative, Acne Support<br>Thomas, Kim; University of Nottingham, School of Medicine, Centre of Evidence Based Dermatology<br>Sach, Tracey; University of East Anglia, Health Economics Group, Norwich Medical School<br>Stanton, Louise; University of Southampton, Southampton Clinical Trials Unit<br>Ridd, Matthew; University of Bristol Faculty of Health Sciences, Population Health Sciences<br>Francis, Nick; University of Southampton, Primary Care Research Centre, Faculty of Medicine, School of Primary Care Population Sciences and Medical Education<br>Little, Paul; University of Southampton, Primary Care Research Centre, Faculty of Medicine, School of Primary Care, Population Sciences and Medical Education<br>Eminton, Zina; University of Southampton, Southampton Clinical Trials Unit<br>Griffiths, Gareth; University of Southampton, Southampton Clinical Trials Unit<br>Layton, Alison M; University of York, Hull York Medical School<br>Santer, Miriam; University of Southampton, Primary Care Research Centre, Faculty of Medicine, School of Primary Care, Population Sciences and Medical Education

**Primary Subject Heading**:	Dermatology
Secondary Subject Heading:	Dermatology
Keywords:	DERMATOLOGY, Acne < DERMATOLOGY, Adult dermatology < DERMATOLOGY

I, the Submitting Author has the right to grant and does grant on behalf of all authors of the Work (as defined in the below author licence), an exclusive licence and/or a non-exclusive licence for contributions from authors who are: i) UK Crown employees; ii) where BMJ has agreed a CC-BY licence shall apply, and/or iii) in accordance with the terms applicable for US Federal Government officers or employees acting as part of their official duties; on a worldwide, perpetual, irrevocable, royalty-free basis to BMJ Publishing Group Ltd ("BMJ") its licensees and where the relevant Journal is co-owned by BMJ to the co-owners of the Journal, to publish the Work in this journal and any other BMJ products and to exploit all rights, as set out in our licence.

The Submitting Author accepts and understands that any supply made under these terms is made by BMJ to the Submitting Author unless you are acting as an employee on behalf of your employer or a postgraduate student of an affiliated institution which is paying any applicable article publishing charge ("APC") for Open Access articles. Where the Submitting Author wishes to make the Work available on an Open Access basis (and intends to pay the relevant APC), the terms of reuse of such Open Access shall be governed by a Creative Commons licence – details of these licences and which Creative Commons licence will apply to this Work are set out in our licence referred to above.

Other than as permitted in any relevant BMJ Author's Self Archiving Policies, I confirm this Work has not been accepted for publication elsewhere, is not being considered for publication elsewhere and does not duplicate material already published. I confirm all authors consent to publication of this Work and authorise the granting of this licence.

For peer review only

Title

Spironolactone for Adult Female Acne (SAFA): A double-blind, placebo-controlled, phase III randomised study of spironolactone as systemic therapy for acne in adult women: study protocol

Names protocol contributors

Susanne Renz[1], Fay Chinnery[1], Beth Stuart[1,2], Laura Day[3], Ingrid Muller[2], Irene Soulsby[4], Jacqueline Nuttall[1], Karen Thomas[4], Kim S Thomas[5], Tracey Sach[6], Louise Stanton[1], Matthew J Ridd[7], Nick Francis[2], Paul Little[2], Zina Eminton[1], Gareth Griffiths[1], Alison M Layton[8], Miriam Santer[2]

Author affiliations

[1]University of Southampton, Southampton Clinical Trials Unit

[2]University of Southampton, Primary Care Research Centre, Faculty of Medicine, School of Primary Care, Population Sciences and Medical Education

[3]Univeristy of Southampton, Southampton Clinical Trials Unit

[4]PPI contributor

[5]Centre of Evidence Based Dermatology, School of Medicine, University of Nottingham

[6]Health Economics Group, Norwich Medical School, University of East Anglia

[7]Population Health Sciences, University of Bristol

[8]Hull York Medical School, University of York

Correspondence: safa@soton.ac.uk, M.Santer@soton.ac.uk

For peer review only - http://bmjopen.bmj.com/site/about/guidelines.xhtml

Abstract

Introduction: Acne is one of the most common inflammatory skin diseases worldwide and can have significant psychosocial impact and cause permanent scarring. Spironolactone, a potassium-sparing diuretic, has anti-androgenic properties, potentially reducing sebum production and hyperkeratinisation in acne prone follicles. Dermatologists have prescribed spironolactone for acne in women for over 30 years, but robust clinical study data are lacking. This study seeks to evaluate whether spironolactone is clinically and cost-effective in treating acne in women.

Methods and analysis: Women (≥18 years) with persistent facial acne requiring systemic therapy, are randomised to receive one tablet daily of 50 mg spironolactone or a matched placebo until week-6, increasing to up to two tablets daily (total 100 mg spironolactone or matched placebo) until week-24, along with usual topical therapy if desired. Study treatment stops at week-24, participants are informed of their treatment allocation and enter an unblinded observational follow-up period for up to 6 months (up to week-52 after baseline). Primary outcome is the Acne-specific Quality of Life (Acne-QoL) symptom subscale score at week-12. Secondary outcomes include: Acne-QoL total and subscales, participant acne self-assessment recorded on a 6-point Likert scale at 6, 12, 24 weeks and up to 52 weeks, Investigator's Global Assessment (IGA) at weeks 6 and 12, cost and cost effectiveness are assessed over 24 weeks. Aiming to detect a group difference of 2 points on the Acne-QoL symptom subscale (s.d. 5.8, effect size 0.35), allowing for 20% loss to follow-up, gives a sample size of 398 participants. **Ethics and dissemination:** This protocol was approved by Wales Research Ethics Committee (18/WA/0420). Follow-up to be completed in early 2022. Findings will be disseminated to participants, peer-reviewed journals, networks and patient groups, on social media, on the study website and the Southampton Clinical Trials Unit (CTU) website to maximise impact.

Study registration: ISRCTN 12892056.

For peer review only - http://bmjopen.bmj.com/site/about/guidelines.xhtml

Strengths and limitations of this study

- Pragmatic design to inform real-world decision making for women with acne includes a primary outcome that is a participant-reported outcome measure, broad eligibility and recruitment strategies via primary care, secondary care, community and social media advertising
- Randomisation to either spironolactone or matched placebo, with participants in both groups using topical treatments as usual (creams, gels, lotions), if desired, in order to reflect the place of oral treatments in the acne care pathway
- Adaptions during the COVID-19 pandemic included inevitable limitations, including remote follow-up visits (via phone or video call), limiting collection of secondary outcomes such as investigator-assessed acne severity

Keywords

Spironolactone; adult female acne; topical therapy; dermatology; randomised controlled study

Background

Acne vulgaris (from here on referred to as acne) is the eighth most common disease worldwide[1] and typically starts in adolescence with 15-20% of people affected showing moderate or severe acne, often persisting into adulthood[2]. Facial scarring occurs in approximately 20% of people but the main impact is social, with levels of psychological disability equivalent to those seen in conditions such as asthma and diabetes[3,4]. Incidence of acne in adult women is considerable and growing[5-9].

First line treatment for acne is topical treatments either alone or combination preparations, containing retinoids, benzoyl peroxide and/or antibiotics[3]. However, non-adherence to topical treatments is common, possibly because of the need to be used consistently for up to 8 weeks and adverse reactions, such as stinging or redness, are common[10]. People therefore commonly seek second-line therapies, such as oral antibiotics, ethinylestradiol/cyproterone acetate (co-cyprindiol) or combined oral contraceptives[3]. In the UK, oral isotretinoin can be used under the supervision of a dermatologist for indications including severe, cystic, nodular or recalcitrant acne. Oral isotretinoin is highly effective, but is not suitable for all patients and is teratogenic[2], therefore needing careful pregnancy prevention management.

A third of people who consult with acne are prescribed long courses of oral antibiotics[3,11]. However, acne is a disease of sebogenesis and antibiotics have no effect on sebum production[12,13]. Furthermore, rising rates of

For peer review only - http://bmjopen.bmj.com/site/about/guidelines.xhtml

antibiotic resistance mean non-antibiotic alternatives are needed[14].

Spironolactone, a potassium-sparing diuretic, is widely used in the UK for indications including hypertension[15] and has been used off-license for women with acne for ≥30 years due to its anti-androgenic properties. US Guidelines suggest a role in the management of female acne[1]. Spironolactone is not used to treat acne in men, because of its feminising side effects[16].

There is limited evidence for the effectiveness of spironolactone for treating acne and the need for evidence from randomised controlled trials (RCTs) in this patient group is acknowledged[16].

When considering spironolactone as a potential alternative systemic therapy for acne, one study reported treatment success in women who had previously failed isotretinoin[17] and a second study comparing the frequency with which participants switched drug treatment within one year of initiation, demonstrated no significant difference between those taking spironolactone and those taking oral antibiotics for their acne,[18] implying they are equally tolerated by users. A further database study has shown that spironolactone may have superior drug usage survival compared to oral antibiotics for women with acne, giving a suggestion of greater perceived effectiveness and tolerability[19].

A James Lind Alliance Priority Setting Partnership, funded by the National Institute for Health Research (NIHR), identified the need to establish the best way to manage acne in women who may or may not have underlying hormonal abnormalities[20]. This informed an NIHR commissioned call (16/13 Persistent acne in adult women) for proposals to answer the research question: 'What is the effectiveness of spironolactone in the treatment of moderate-severe persistent acne in adult women?'

This study aims to answer whether spironolactone in addition to standard topical therapy is able to improve acne-related quality of life in adult women with moderate-severe persistent facial acne compare to placebo plus standard topical therapy.

Methods

Study design and setting

The study is a phase III, multicentre, double-blind, randomised superiority study, to investigate clinical and cost-effectiveness of spironolactone in the treatment of moderate or severe persistent facial acne in adult women compared to placebo, in addition to standard topical treatment. The design is pragmatic in order to have strong external validity and to inform real-world decision-making for women with acne and their health professionals. Pragmatic design includes broad eligibility and recruitment strategies, a primary outcome that

For peer review only

For peer review only - http://bmjopen.bmj.com/site/about/guidelines.xhtml

is relevant to participants, low intensity follow-up and an intention-to-treat (ITT) analysis. 'Moderate to severe acne', in the context of this study, is defined as acne that warrants treatment with oral antibiotics, as judged by the potential participant and study clinician.

Baseline and follow-up appointments are carried out by UK hospital dermatology centres in order to facilitate blood tests at baseline and clinical assessments. Participants continue on the allocated treatment (spironolactone or placebo) in combination with their usual topical treatment (if desired) for a total duration of 24 weeks with assessments at weeks 6 and 12. Primary outcome is assessed at week-12 with the patient-reported Acne-QoL[21,22]. From week-12, participants in both groups may receive 'usual care' from their usual clinical team including oral treatments (oral antibiotics, hormonal treatments), and from week-24 isotretinoin, if the participant and study clinician feel the need for rescue treatment. Trial participants receive shopping vouchers at baseline, 6 and 12 weeks (total £40). At week-24, participants stop taking their study treatment, are informed of their treatment group allocation and enter an unblinded observational follow-up period for up to six months (up to week-52 after baseline). During this observational final follow-up period, participants can ask their General Practitioner (GP) to be prescribed spironolactone for their acne if they wish, or pursue other acne treatments as part of usual care. Figure 1 illustrates the patient pathway through the study with Table 1 summarising the study procedures.

Adaptations during the COVID-19 pandemic included the option to hold follow-up visits at weeks 6 and 12 remotely (phone/video calls) and to post out the study tablets and questionnaires to participants. Participants were also given standardised instructions on how to photograph their face to submit as part of remote follow-up assessments. Baseline visits continued to be face-to-face due to the requirement of a urine pregnancy test and blood test to assess kidney function and serum potassium levels.

FIGURE 1: STUDY SCHEMA

TABLE 1: SCHEDULE OF PROCEDURES

Eligibility

Inclusion criteria

Participants should fulfil all the following criteria:

- Women aged ≥18 years
- Facial acne vulgaris, symptoms present for at least 6 months
- Acne of sufficient severity to warrant treatment with oral antibiotics, as judged by the study clinician.

For peer review only - http://bmjopen.bmj.com/site/about/guidelines.xhtml

Patients with an IGA≥2 are eligible to participate in the study

- Women of childbearing potential at risk of pregnancy must be willing to use their usual hormonal or barrier method of contraception for the first 6 months of the study (whilst taking the study investigational medicinal product (IMP)) and for at least 4 weeks afterwards
- Willing to be randomised to either study group
- Willing and able to give informed consent
- Sufficient English to carry out primary outcome Acne-QoL

Exclusion criteria

Individuals meeting any of the following criteria will be excluded:

- Acne grade 0-1 using IGA (i.e. clear or almost clear)
- Has ever taken spironolactone
- Oral antibiotic treatment (lasting longer than a week) for acne within past month
- Oral isotretinoin treatment within past 6 months
- Started, stopped or changed long-term (lasting more than 2 weeks) hormonal contraception, ethinylestradiol/cyproterone acetate (co-cyprindiol) or other hormonal treatment within past 3 months
- Planning to start, stop or change long-term (lasting more than 2 weeks) hormonal contraception, ethinylestradiol/cyproterone acetate (co-cyprindiol) or other hormonal treatment within the next 3 months
- Pregnant/breastfeeding
- Intending to become pregnant in the next 6 months
- Contra-indicated to spironolactone:
    - Currently taking potassium-sparing diuretic, ACE inhibitor, angiotensin II receptor blocker or digoxin
    - Hereditary problems of galactose intolerance, lactase deficiency or glucose-galactose malabsorption (as the spironolactone and placebo tablets contain lactose)
    - Androgen-secreting adrenal or ovarian tumour
    - Cushing's syndrome
    - Congenital adrenal hyperplasia
    - Estimated Glomerular Filtration Rate (eGFR) below 60 ml/min/1.73m^2

For peer review only

For peer review only - http://bmjopen.bmj.com/site/about/guidelines.xhtml

- Serum potassium level above upper limit of reference range for the laboratory processing the sample

Intervention and control

Study participants receive one tablet daily (50 mg spironolactone or matched placebo) for the first six weeks of the study. At, or any time after the week-6 visit, the dose is escalated to two tablets daily (total 100 mg spironolactone or matched placebo) by the study clinician, providing the participant is tolerating any side effects (see box 1). Participants are instructed to take their total dose once daily in the morning to avoid diuresis later in the day/evening. All known adverse effects of spironolactone are detailed in the Patient Information Sheet and trial participants have the opportunity to discuss the trial with the site team and discuss any questions before consenting to enter the trial.

Participants may use their usual topical treatments throughout the study but adherence to topicals is not actively promoted as this may mask differences between the randomised groups. Participants are discouraged from changing their topical treatments between baseline and week-12.

INSERT BOX 1: RATIONAL FOR DOSING REGIMEN

Intervention adherence

The hospital study team assess participant adherence to treatment at each study visit using pill counts. In cases where only remote visits are possible, the participant informs the study team of the number of tablets remaining. However, no additional support or activity is undertaken to encourage daily pill taking, in keeping with the study's pragmatic design.

Randomisation and assignment to intervention group

Following consent, participants are randomised 1:1 by clinical staff at the hospital site using an independent web-based system (TENALEA) using varying blocks of size 2 and 4, stratified by centre and by baseline severity (IGA<3 vs 3 or more). The allocation sequence was generated by a statistician using computer-generated random numbers. Participants, study staff and investigators are blind to the treatment allocation until end of the treatment period at week-24. Treatment allocation is revealed prior to week-24 only if there is a clinical need to do so.

Recruitment

Potential participants, identified in primary and secondary care, direct advertising in areas close to recruiting

For peer review only

For peer review only - http://bmjopen.bmj.com/site/about/guidelines.xhtml

hospitals and social media advertising, are directed to the study website (https://www.southampton.ac.uk/safa) or to contact the local study team directly.

In primary care, recruitment is supported by general practices acting as Participant Identification Centres (PIC) local to the recruiting centres identifying potential participants either opportunistically or via database search based on an acne diagnosis and relevant prescription within the past 6 months and mail-out of invitation pack. In secondary care, potential participants are identified opportunistically in out-patient clinics and through screening new referral letters.

We use targeted social media advertising to promote the study, build study awareness and interest.

Participants are free to withdraw consent from the study at any time without providing a reason. They may withdraw from study treatment but remain in follow-up; withdraw from study and follow-up, but give permission for their data to be used in analyses; or completely withdraw from the study and not permit their data to be used.

Primary and secondary outcome measures

Clinically, the effectiveness of acne treatments is usually judged at 8-12 weeks, so the primary outcome in the study is assessed at week-12 with the Acne-QoL symptom subscale score[21,22]. The Acne-QoL was developed and validated for use in a clinical study to assess the impact of therapy on quality of life among people with facial acne and the primary outcome at week-12 is the symptom subscale score of the Acne-QoL, because the Acne-QoL was intended to be presented as 4 separate subscale scores. It was not designed or validated to have a total score, however, it has published Minimum Clinically Important Difference (MCID) of 2 points for the subscales and range 0 to 30 for symptom subscale score[21-23]. Other participant-reported outcome measures in acne do not have a published MCID available and have not been found to have advantages in terms of acceptability and validity [24].

Secondary outcomes include Acne-QoL at week-24, participant self-assessed overall improvement recorded on a 6-point Likert scale with photographs taken at baseline[25], IGA[5], participant global assessment, use of acne medication and participant satisfaction with study treatment. Trial participants are at each time point asked which topical or oral treatments were used in the period since the previous time point (if any) and have the option to add more information of the treatments for their acne in a free text box of the participant questionnaires. The IGA is a 5-point scale ranging from clear to severe (0 ‘Clear’; 1 ‘Almost clear’; 2 ‘Mild’; 3 ‘Moderate’; 4 ‘Severe’)[6,26]. The IGA[5,25] is used to grade the participant’s acne as lesion counts are time

For peer review only - http://bmjopen.bmj.com/site/about/guidelines.xhtml

consuming, with wide inter-assessor variation and give little additional information to global assessments. All outcome measures are shown in Table 2.

The safety profile of spironolactone is well established[15,16]. Consequently, we collect information about adverse reactions of special interest, both to inform the dose review decision from week-6 onwards as well as to learn more about incidence of side effects in this population. We also collect and report all serious adverse events (SAEs).

TABLE 2: SCHEDULE OF OBSERVATIONS

Pregnancy

Spironolactone is considered contraindicated in pregnancy, or a category C drug (i.e. potential benefits may warrant use in pregnant women despite potential risks)[1,15]. The main concern is around possible feminisation of the male foetus in the third trimester of pregnancy[1]. Women of childbearing potential at risk of pregnancy will be asked to use their usual hormonal or barrier method of contraception during the first 24 weeks of the study and for at least 4 weeks (approximately one menstrual cycle) afterwards. A pregnancy test will be conducted for all participants at their baseline visit and documented in their medical notes. At weeks 6 and 12, the study nurse/doctor will ask participants to confirm that they are still using contraception and have not changed contraceptive method. Participants who become pregnant will be asked to inform their local site study team as soon as possible and will not be able to continue in the study.

Sample size

Based on comparison of the Acne-QoL symptom score between groups at week-12, power 90%, alpha 0.05 and seeking a difference between groups of 2 points on the symptom subscale (s.d. 5.8, effect size 0.35), 346 participants are needed. Allowing for 20% loss to follow up gives a total 434 participants (217 per group). Following discussions with oversight committees post funding award, the sample size was recalculated. Allowing for a correlation with baseline of 0.293 and a deflation factor of 1-ρ2[27], gives a total sample size required of 398 participants. A difference of 2 points on the symptom subscale and a standard deviation of 5.8 (equivalent to an effect size 0.35) is in line with that reported in studies in a similar patient group and with the MCID reported for Acne-QoL[21,22].

Data collection methods

For peer review only - http://bmjopen.bmj.com/site/about/guidelines.xhtml

Participant data are entered into study electronic case report forms (eCRF) via a remote data collection tool (Medidata Rave) by trained hospital research personnel with specific roles on the study and regularly checked for missing or anomalous values by Southampton CTU study staff.

Data management

Participant data are pseudonymised by assigning each participant a participant identifier code, which is used to identify the participant during the study and for any participant-specific communication between Southampton CTU and recruiting sites. The site retains a participant identification code list which is only available to site staff and stored in a secure location at site.

Patient and public involvement (PPI)

This study addresses a priority area identified as important to patients and health professionals by the James Lind Alliance Priority Setting Partnership for Acne[7]. We gained feedback from a virtual acne-specific patient panel, convened through 'People in Research' (https://www.peopleinresearch.org). A patient survey was carried out with the support of the UK Dermatology Clinical Trials Network (UK DCTN) in order to inform the study design. Findings suggested that participants would find it difficult to abstain from using topical treatments for more than 12 weeks and that asking participants to take a placebo for one year would also be a barrier to recruitment.

Two public contributors with experience of acne attend all Trial Management Group (TMG) meetings to ensure that decisions around study design are informed by their perspective, study procedures are feasible for participants and study materials are readable and include all the relevant information that participants would want. Public contributors influenced the trial design and delivery, for instance by advocating use of social media advertising to improve recruitment, arguing against repeated measures in this patient group and that an upper age limit of 50 years was arbitrary and could appear discriminatory.

Statistical methods

The study will be reported in accordance with CONSORT guidelines. A detailed statistical analysis plan (SAP) will be written and reviewed prior to the study database being locked.

The modified ITT population consists of all participants who have consented and been randomised to a treatment arm and have complete data for the outcome being analysed. Analyses will be performed according to the modified ITT principle using a linear regression model. All analyses will be carried out in the modified ITT population, with the level of missing data reported, unless otherwise stated. The frequency and

For peer review only - http://bmjopen.bmj.com/site/about/guidelines.xhtml

pattern of missing data will be examined and a multiple imputation model will be used as a sensitivity analysis if appropriate.

For the primary analyses, descriptive statistics will be obtained for the randomised groups to characterise recruited participants and assess baseline comparability. For the primary outcome, a linear regression model will be used to analyse Acne-QoL symptom subscale at week-12, adjusting for baseline variables (including baseline Acne-QoL symptom subscale score, use of topical treatments, use of hormonal contraception/ ethinylestradiol/cyproterone acetate (co-cyprindiol) and randomisation stratification variables (centre, baseline severity (IGA < 3 versus 3 or more)). A full list of covariates and model specification will be set out in the SAP. A 95% confidence interval for the least squares mean difference between arms in Acne-QoL symptom subscale at week-12 will be calculated.

The same analysis methods will be used to summarise Acne-QoL symptom subscale at other time points (weeks 6, 24 and up to week-52eeks after baseline) and for the other Acne-QoL subscales (self-perception, role-emotional and role-social) and total score. IGA and participants' comparison with baseline photo at weeks 6, 12 and 24 will be dichotomised as success or failure as recommended by the US Food and Drug Administration (with success for IGA and participants global assessment defined as clear or almost clear (grade 0 or 1) and at least a two-grade improvement from baseline; this represents a clinically meaningful outcome). The dichotomised outcomes will be summarised by frequencies and percentages and compared by group using logistic regression adjusting for baseline assessment, use of hormonal contraception/ ethinylestradiol/cyproterone acetate (co-cyprindiol), use of topical treatment and randomisation stratification factors.

Adverse reactions of special interest and SAEs will be summarised by group with frequencies and percentages and compared with Pearson's χ^2 tests. Logistic regression modelling will also be used to adjust for any important differences in topical treatment use by group. Subgroup analyses will investigate how the treatment effect differs by whether participants have symptoms consistent with polycystic ovary syndrome (PCOS) as recorded at the baseline visit. It is acknowledged the study is not powered for this subgroup analysis. The same analysis methods will be applied to the outcomes collected at up to 52 weeks however the interpretation of these results will be assessed with caution as participants will potentially have been off treatment for up to 6 months or started a different acne treatment. All analyses will be carried out using STATA and/or SAS.

There are no planned interim analyses or subgroup analyses.

For peer review only - http://bmjopen.bmj.com/site/about/guidelines.xhtml

Health economics analysis

If the intervention is found effective, a within-study economic evaluation will be undertaken to assess value for money of spironolactone plus usual care versus placebo plus usual care. The main perspective of the analysis will be that of the NHS over the 24 week treatment period, although a secondary analysis will assess the importance of a broader perspective by incorporating out of pocket costs related to acne and any productivity/employment impacts for people with persistent acne[28].

Costs, including intervention and wider NHS resource use, are being recorded in the study eCRF for the former whilst wider NHS resource use is captured in participant questionnaires at baseline, weeks 6, 12, and 24. Costs will be valued using published unit costs for a common recent price year to estimate mean cost per participant in each arm[29-31].

Review of the reliability, validity and responsiveness of three generic preference-based measures (EQ-5D, SF-6D and HUI) in skin conditions only found evidence to support the use of the EQ-5D in skin diseases with no studies looking at measurement properties for the Short-Form six-dimensions (SF-6D) or Health Utilities Index (HUI) in skin disease[32]. Problems on the EQ-5D domains were found to be substantially higher in the acne sample receiving specialist care than in an age truncated population sample (aged 20-39 years) particularly on the pain/discomfort (42.1% in the acne sample versus 17.7% in an age-truncated population sample) and anxiety/depression domains (52.8% versus 12.5% respectively)[33]. EQ-5D was found to be responsive to change, with moderate effect sizes at 4 and 12 months (-0.44 and -0.53 respectively)[33]. We will value the EQ-5D-5L in our primary analysis in line with NICE recommendations at the time of analysis[3,34]. Quality Adjusted Life Years (QALYs) (estimated using EQ-5D-5L[32,33]) for the study period will be estimated using linear interpolation and area under the curve with and without baseline adjustment[35]. Clinical measures were found to be more responsive to change than the generic measures (shown by larger effect sizes) and combination of generic preference based measures with the use of disease specific measure was concluded to be desirable[33]. The primary economic evaluation will be an incremental cost utility analysis to enable the cost effectiveness to be compared across a range of health conditions and interventions such that decision makers can use the information to inform prioritisation of health care. A secondary cost effectiveness analysis using the disease specific Acne-QoL will be presented as appropriate, though it should be noted that this instrument does not have utility weights available and it is unclear what incremental cost per unit of change on the Acne-QoL represents good value for money. All analysis will be conducted and presented using established methods[28,36].

For peer review only - http://bmjopen.bmj.com/site/about/guidelines.xhtml

If spironolactone is not found to be clinically effective a full economic evaluation will not be conducted. Instead, estimates of mean costs and utility per participant will be presented at the various study time points as these may be informative for other researchers undertaking future economic studies or economic modelling in this clinical area.

A detailed Health Economic Analysis Plan will be written prior to the study database being locked.

Oversight and monitoring

The TMG includes representatives with expertise in dermatology, primary care research, psychology, medical statistics and health economics, public contributors, and Southampton CTU staff involved in the day-to-day running of the study and is responsible for the oversight of the progress of the study. An independent Trial Steering Committee (TSC) and an independent Data Monitoring and Ethics Committee (DMEC) have been set up to monitor study progress and safety.

Data on adverse reactions will be collected at the baseline and follow-up visits, and participants will be asked to report any adverse reactions in their week-24 questionnaire. SAEs may be identified by participant report at any time directly to the hospital study team, at follow-up visits or questionnaires. Participants' GPs will be informed of their patient's participation in the study and asked to notify the hospital study team of any potential SAE. The study also has a UK regulatory compliant real-time SAE reporting process to identify serious adverse reactions and suspected unexpected serious adverse reactions that could suspend or stop the study if warranted.

The Southampton CTU has undertaken a risk assessment for the study which includes the requirements for monitoring (both central and site). The Southampton CTU undertakes a number of internal audits of its own systems and processes annually and has routine audits from both its sponsor and the independent Medicine and Health care products regulatory authority (MHRA) every 2-3 years.

Discussion

This is the first adequately powered pragmatic, randomised trial investigating the effect of spironolactone on acne in adult women in comparison to a matched placebo. If found to be clinically and cost effective, use of spironolactone will likely become the new standard of care in addition to topical treatments potentially reducing antibiotic use for women requiring systemic therapy.

For peer review only - http://bmjopen.bmj.com/site/about/guidelines.xhtml

Respondents to a survey of people with experience with acne reported that they would be unwilling to be recruited to a study where they remained blinded to the treatment allocation for 52 weeks, due to concerns about potential worsening of acne over this time. Therefore, the study was designed as blinded treatment phase of 24 weeks with an observational follow-up period for up to 6 months after. Use of acne treatments, such as oral isotretinoin or antibiotics, between week-24 and up to week-52 are carefully recorded as differences between groups and would be important in interpreting week-52 outcomes. Use of topical treatments is allowed in both groups during the 24 week treatment phase as i) women with moderate-severe acne may be unwilling to be randomised to placebo alone, and ii) recruiting women with moderate-severe acne to a placebo-controlled study with no effective treatment for 12 weeks in the control arm may risk worsening of acne and possible scarring.

Although others are seeking to evaluate the role of spironolactone in comparison with oral tetracyclines[37], this is the largest study to date to inform clinical practice over the effectiveness of spironolactone as an alternative treatment for acne in adult women.

Abbreviations

ACE	Angiotensin-converting enzyme
CRN	Clinical Research Network
CTU	Clinical Trials Unit
DMEC	Data Monitoring and Ethics Committee
eCRF	Electronic Case Report Form
eGFR	Estimated Glomerular Filtration Rate
EQ-5D-5 L	EuroQol Five Dimensions Five Level
GP	General Practitioner
HUI	Health Utilities Index
HRA	Health Research Authority
HTA	Health Technology Assessment

For peer review only

For peer review only - http://bmjopen.bmj.com/site/about/guidelines.xhtml

ICF	Informed Consent Form
IGA	Investigator’s Global Assessment
IMP	Investigational Medicinal Product
IPD	Individual Patient Data
ITT	Intention-to-treat
MCID	Minimum Clinically Important Difference
MHRA	Medicines and Healthcare products Regulatory Agency
NHS	National Health Service
NICE	National Institute for Health and Care Excellence
NIHR	National Institute for Health Research
PCOS	Polycystic Ovary Syndrome
PGA	Participant’s Global Assessment
PIC	Participant Identification Centre
PIS	Participant Information Sheet
PPI	Patient and Public Involvement
PSS	Personal Social Services
QALYs	Quality Adjusted Life Years
QoL	Quality of Life
RCT	Randomised Controlled Trial
REC	Research Ethics Committee
SAE	Serious Adverse Event
SF-6D	Short Form Six Dimensions
SpR	Specialty registrar

For peer review only

For peer review only - http://bmjopen.bmj.com/site/about/guidelines.xhtml

TMG	Trial Management Group
TSC	Trial Steering Committee
UHS	University Hospital Southampton NHS Foundation Trust
UK DCTN	UK Dermatology Clinical Trials Network

Declarations

Acknowledgements

- The virtual patient panel (brought together by NIHR INVOLVE) for their advice on the study design.
- The study was developed with support from the UK Dermatology Clinical Trials Network (UK DCTN). The UK DCTN is grateful to the British Association of Dermatologists and the University of Nottingham for financial support of the Network. UK DCTN conducted surveys among patients with acne and health professionals managing acne to inform the study design, promoting the study and identifying and advising on potential hospital sites delivering the study.
- Wessex CRN for funding the initial social media advertising campaign.
- Jessica Boxall and Liz Allaway for management of the study social media accounts as well as running and coordinating the social media adverts.
- Hospital dermatology centres recruiting for the study: Queen Elizabeth Hospital, Birmingham; Bristol Royal Infirmary Dermatology Centre, Bristol; University Hospital of Wales, Cardiff; General Hospital, Epsom; District Hospital, Harrogate; St Mary’s Hospital (Imperial College NHS Healthcare Trust), London; Queen’s Medical Centre, Nottingham; General Hospital, Poole; St Mary’s General Hospital Dermatology Centre, Portsmouth; Swansea Bay University Health Board, Swansea.
- Participant Identification Centres (PICs) for searching their patient lists and mail outs and Clinical Research Networks (CRNs) for helping to identify potential PICs.

Authors’ contributions

Research funding was obtained by MS, AL, NF, GG, PL, IM, JN, MJR, TS, IS, LS, BS, KT and KST. All authors have contributed to the development of the protocol and to the management of the study. SR leads on the day-to-day management of the study, overseen by ZE, MS, JN and BS. This paper was drafted by FC

For peer review only

For peer review only - http://bmjopen.bmj.com/site/about/guidelines.xhtml

and SR with contributions from LD, MS and all authors. All authors read and approved the final manuscript.

Funding

This project is funded by the National Institute for Health Research (NIHR) Health Technology Assessment (HTA) programme (Grant Reference Number: 16/13/02) and supported by NIHR CTU support funding at Southampton CTU. The views expressed are those of the author(s) and not necessarily those of the NIHR or the Department of Health and Social Care. The NIHR HTA funder will play no role in the execution, analysis, interpretation of data, or study publication. The study is registered on the UK NIHR study portfolio meaning there are research nurses based at UK hospitals who help in screening potential patients to identify those eligible for the study.

Southampton CTU, a NIHR CTU support funded UK Clinical Research Collaboration registered CTU, is coordinating the study. University of Southampton is the sponsor for the study.

Trial status

This clinical trial was registered on 17-Sep-2018 (EudraCT Number: 2018-003630-33) and on 15-Oct-2018 (ISRCTN, registry number: ISRCTN 12892056). The first participant was recruited on 05-Jun-2019 and recruitment is expected to be completed on 31-Aug-2021. The current protocol is version 10, dated 08-Mar-2021. The full protocol v10 08-MAR-2021 is available as supplementary material and on request via safa@soton.ac.uk or on the study website https://www.southampton.ac.uk/safa/about/researchers-and-clinicians.page. REC/MHRA approved protocol amendments will be communicated to sites via email and updated trial documentation provided centrally via the trial website. Trial registries will be amended where relevant with explanations for these changes. End of study is defined as the date when the last point of data is collected for the last participant from their Final follow-up questionnaire.

Ethics approval

The trial received favourable ethical opinion from Wales Research Ethics Committee (18/WA/0420) and has Health Research Authority (HRA) approval (IRAS 246637).

Availability of data and materials

Data Management Plan

Full details of the data management strategy for the study are available in the SAFA data management plan,

For peer review only - http://bmjopen.bmj.com/site/about/guidelines.xhtml

available on request via safa@soton.ac.uk.

Underlying data

Pseudonymised individual participant data (IPD) within the clinical study dataset will be available for sharing via controlled access by authorised Southampton CTU staff (as delegated to Southampton CTU by the study sponsor). Data access can be requested via a Southampton CTU Data Release application form (available from https://www.southampton.ac.uk/ctu/about/index.page) after the trial is published. Please email the completed form to the Southampton CTU Data Release Committee Coordinator at ctu@soton.ac.uk.

Data access requests are reviewed against specific eligibility criteria by the Southampton CTU data custodian and key members of the study team, including a statistician and chief investigator or by an external Independent Review Panel. Decisions about requests are made promptly and usually no more than three months after receipt of request. Responses to all data requests, with a clear rationale for any refusals, will be sent promptly to the data requester.

Extended data

Written informed consent is obtained from participants during the baseline visit by qualified site trial team members. A copy of the study's Informed Consent Form (v4 05-MAR-2021) is available as supplementary material or on request via safa@soton.ac.uk .

Consent for publication

Responsibility for publication has been delegated to Prof Miriam Santer and Prof Alison Layton (co-Chief Investigators) and Prof Gareth Griffiths (Director of Southampton CTU), who have consented to this publication.

Competing interests

The authors have no competing interests to declare.

Authors' information (optional)

Not applicable.

Tables

Table 1: Schedule of procedures

For peer review only

For peer review only - http://bmjopen.bmj.com/site/about/guidelines.xhtml

**Observation/procedure**	**Person undertaking the specified event**	**Visit**				
		Screening/ Baseline	*Week 6*	*Week 12*	*End of Treatment/ Week 24 contact*	*End of Study/ Final Follow-up questionnaire (52 weeks or sooner)*[3]
**ENROLMENT**						
Informed Consent	Nurse/ Other clinician[1]	X				
Eligibility evaluation	Other clinician	X				
Participant characteristics	Nurse/Other clinician	X				
Blood pressure	Nurse/Other clinician	X				
Blood tests (serum potassium, eGFR)	Nurse/Other clinician	X				
Pregnancy test	Nurse/Other clinician	X				
**RANDOMISATION**	Nurse/Other clinician	X				
**ASSESSMENTS**						
Investigator's Global Assessment (IGA)	Nurse/ Other clinician	X	X	X		
Medical history	Participant	X				
Self-assessment - Participants Global Assessment (adapted IGA)	Participant	X	X	X	X	X
Acne Medication Use	Nurse/Other clinician/Participant	X	X	X	X	X
Other Medication Use	Nurse/Other clinician/Participant	X				
Acne-QoL	Participant	X	X	X	X	X
EQ-5D-5L	Participant	X	X	X	X	X
Resource use questionnaire	Participant	X	X	X	X	X
Self-assessment – comparison with baseline photo - 6 Point Likert Scale	Participant		X	X	X	X
Collection of ARs of special interest:<br>Headache<br>Dizziness/vertigo/ Light headedness<br>Tingling<br>Indigestion/heartburn/ Dyspepsia<br>Diarrhoea<br>Polyuria (passing much more urine than normal)<br>Nausea/feeling sick<br>Vomiting/being sick<br>Tenderness of the breasts<br>Breast enlargement<br>Irregular menstrual periods<br>Abdominal pain<br>Weight gain<br>Reduced libido (reduced interest in sex)<br>Fatigue/tiredness<br>Drowsiness/sleepiness	Participant/ Other clinician		X	X	X	
Serious Adverse Events	Other clinician (PI or delegate)		X	X	X	

For peer review only

For peer review only - http://bmjopen.bmj.com/site/about/guidelines.xhtml

**Observation/procedure**	**Person undertaking the specified event**	**Visit**				
		Screening/ Baseline	*Week 6*	*Week 12*	*End of Treatment/ Week 24 contact*	*End of Study/ Final Follow-up questionnaire (52 weeks or sooner)*[3]
Assessment of treatment response to determine dose adjustment[2]	Participant / Other clinician		X	X		
Satisfaction with study treatment	Participant				X	
**OTHER ACTIVITIES**						
Discuss use of contraception	Nurse/Other clinician	X	X	X		
Photographs of face taken	Nurse/Other clinician	X				
Photographs given to participant	Nurse/Other Clinician	X		X (If a set was stored at site)		
Letter to participant's GP (patient participation)	Nurse/Other clinician	X				
Check participant is not using oral acne treatment	Nurse/Other clinician/Participant		X	X		
Return excess IMP to clinic	Participant		X	X	X (return via post)	
Spironolactone/Placebo pill count	Nurse/Other clinician/Participant		X	X	X	
Letter to participant's GP (if dose is change)	Nurse/Other clinician		X	X		
Reminder to participant to report any subsequent adverse event(s) that might reasonably be related to participation in this study (up to 52 weeks)	Nurse/Other clinician			X		
Ask participant if they would like to receive a summary of the study results, when available	Nurse/Other clinician			X		
Letter to participant (unblinding)					X (after 24 weeks)	
Letter to participant's GP (unblinding)					X (after 24 weeks)	

[1] Dermatologist or Clinical Research Fellow, in line with local procedures with demonstrable and appropriate level of training. Specific duties delegated by the PI and listed on the delegation log.
[2]Dose escalated to 2 tablets per day if participant is tolerating side effects.
[3] The follow-up questionnaire will be sent out 6 months or sooner after the 24-week timepoint.

Table 2: Schedule of observations

For peer review only - http://bmjopen.bmj.com/site/about/guidelines.xhtml

Outcome measure	6 weeks	12 weeks (primary endpoint)	24 weeks (end of treatment)	Unblinded Follow-up (52 weeks or sooner)[h]
**Primary outcome measure**				
Acne-QoL symptom subscale score		X		
**Secondary outcome measures**				
Acne-QoL symptom subscale score	X	X	X	X
Acne-QoL other subscales[a]	X	X	X	X
Acne-QoL total score	X	X	X	X
Participant self-assessed overall improvement[b]	X	X	X	X
Investigator's Global Assessment (IGA)[c]	X	X		
Participant's Global Assessment (PGA)[d]	X	X	X	X
Participant satisfaction with study treatment[e]			X	
Health-related quality of life using EQ-5D-5L[f]	X	X	X	X
Costs incurred	X	X	X	X
Cost-effectiveness[g]			X	

505 [a] Self-perception, role-emotional and role-social; [b] recorded on a 6-point Likert scale with photographs taken at the baseline visit to aid
506 recall[37]; [c] 5-point scale ranging from clear to severe (0 - Clear; 1 - Almost clear; 2 - Mild; 3 - Moderate; 4 - Severe)[38];
507 [d] Same scale as the IGA, but written in Plain English for participants' use; [e] Asked prior to revealing treatment allocation after 24 weeks;
508 [f] The EQ-5D-5 L assesses five dimensions: mobility, self-care, usual activities, pain/discomfort and anxiety/depression; [g] Using EQ-5D-
509 5L and data on health resource use during the study; [h] The follow-up questionnaire will be sent out 6 months or sooner after the 24-
510 week timepoint.

517 **BOX 1**

For peer review only - http://bmjopen.bmj.com/site/about/guidelines.xhtml

For peer review only

Rationale for the dosing regimen

We conducted a survey of health professionals to inform the spironolactone dose regimen (unpublished). Responses were received from 41 Dermatology consultants, 10 Dermatology nurses and 3 Dermatology SpRs.

Of these 54 Dermatology health professionals, 22 prescribed spironolactone (9 rarely, 10 sometimes and 3 often). Most of those who responded stated that they would start at 50mg and increase up to 100-150mg depending on response. Several noted that this would depend on the patient's weight, with the starting dose lowered to 25mg if needed and allowing the dosage to increase up to 200mg. There was no consistency on the timeframe for these increases with 4 weeks, 6 weeks, 12 weeks and 6 months all being mentioned as review points.

A previous HTA study examining common treatments in the management of acne suggested that assessing efficacy at 6 weeks was ideal[25] – this informed the timing of follow-up assessments and dose escalation. US guidelines note that studies have been carried out using spironolactone doses ranging from 50mg to 200mg daily.[1] No specific dose is recommended but it is noted that side effects are dose-related.[1]

A recent hybrid systematic review of RCTs and case series identified some very low-quality evidence which showed that a daily dose of 200mg was statistically significantly more effective than placebo versus inflamed lesions, but it also confirmed that this dose is associated with a significantly greater risk of adverse side effects than lower doses.[17]

Hence, there would appear to be no merit in using these higher doses for managing acne. Data from the multiple case series suggested that any future RCT examining lower doses is likely to generate results that confirm the effectiveness and better safety profile of doses ≤100 mg per day, which informed the dosage regimen.

For most licensed indications for spironolactone, the British National Formulary states a starting dose of 100mg, titrated as required. Therefore, a starting does of 50mg in the SAFA study seems conservative.

References

For peer review only - http://bmjopen.bmj.com/site/about/guidelines.xhtml

1. Zaenglein AL, Pathy AL, Schlosser BJ, et al. Guidelines of care for the management of acne vulgaris. *J Am Acad Dermatol* 2016;74(5):945-73.e33.
2. Williams HC, Dellavalle RP, Garner S. Acne vulgaris. *The Lancet* 2012;379(9813):361-72.
3. NICE. Clinical Knowledge Summaries: Acne vulgaris. (accessed 01 July 2019).
4. Tan J, Kang S, Leyden J. Prevalence and Risk Factors of Acne Scarring Among Patients Consulting Dermatologists in the USA. *J Drugs Dermatol* 2017;16(2):97-102.
5. Holzmann R, Shakery K. Postadolescent acne in females. *Skin Pharmacol Physiol* 2014;27 Suppl 1:3-8.
6. Kim GK, Michaels BB. Post-adolescent acne in women: more common and more clinical considerations. *J Drugs Dermatol* 2012;11(6):708-13.
7. Perkins AC, Maglione J, Hillebrand GG, et al. Acne vulgaris in women: prevalence across the life span. *J Womens Health (Larchmt)* 2012;21(2):223-30.
8. Tan JK, Li Y, Fung K, et al. Divergence of demographic factors associated with clinical severity compared with quality of life impact in acne. *J Cutan Med Surg* 2008;12(5):235-42.
9. Lynn DD, Umari T, Dunnick CA, et al. The epidemiology of acne vulgaris in late adolescence. *Adolesc Health Med Ther* 2016;7:13-25.
10. Thiboutot D, Dréno B, Layton A. Acne counseling to improve adherence. *Cutis* 2008;81(1):81-6.
11. Francis NA, Entwistle K, Santer M, et al. The Management of Acne Vulgaris in Primary Care: A cohort study of consulting and prescribing patterns using CPRD. *British Journal of Dermatology* 2016:n/a-n/a.
12. Del Rosso JQ, Webster GF, Rosen T, et al. Status Report from the Scientific Panel on Antibiotic Use in Dermatology of the American Acne and Rosacea Society: Part 1: Antibiotic Prescribing Patterns, Sources of Antibiotic Exposure, Antibiotic Consumption and Emergence of Antibiotic Resistance, Impact of Alterations in Antibiotic Prescribing, and Clinical Sequelae of Antibiotic Use. *J Clin Aesthet Dermatol* 2016;9(4):18-24.
13. Khondker L, Khan SI. Acne vulgaris related to androgens - a review. *Mymensingh Med J* 2014;23(1):181-5.
14. Walsh TR, Efthimiou J, Dréno B. Systematic review of antibiotic resistance in acne: an increasing topical and oral threat. *Lancet Infect Dis* 2016;16(3):e23-33.
15. Formulary BN. *British Medical Association and the Royal Pharmaceutical Society*. http://www.bnf.org.
16. Layton AM, Eady EA, Whitehouse H, et al. Oral Spironolactone for Acne Vulgaris in Adult Females: A Hybrid Systematic Review. *American journal of clinical dermatology* 2017;18(2):169-91.
17. Isvy-Joubert A, Nguyen JM, Gaultier A, et al. Adult female acne treated with spironolactone: a retrospective data review of 70 cases. *Eur J Dermatol* 2017;27(4):393-98.
18. Barbieri JS, Choi JK, Mitra N, et al. Frequency of Treatment Switching for Spironolactone Compared to Oral Tetracycline-Class Antibiotics for Women With Acne: A Retrospective Cohort Study 2010-2016. *J Drugs Dermatol* 2018;17(6):632-38.
19. Barbieri JS, Choi JK, James WD, et al. Real-world drug usage survival of spironolactone versus oral antibiotics for the management of female patients with acne. *J Am Acad Dermatol* 2019;81(3):848-51.
20. Layton A, Eady EA, Peat M, et al. Identifying acne treatment uncertainties via a James Lind Alliance Priority Setting Partnership. *BMJ Open* 2015;5(7):e008085.
21. Fehnel SE, McLeod LD, Brandman J, et al. Responsiveness of the Acne-Specific Quality of Life Questionnaire (Acne-QoL) to treatment for acne vulgaris in placebo-controlled clinical trials. *Qual Life Res* 2002;11(8):809-16.
22. McLeod LD, Fehnel SE, Brandman J, et al. Evaluating minimal clinically important differences for the acne-specific quality of life questionnaire. *PharmacoEconomics* 2003;21(15):1069-79.
23. Martin AR, Lookingbill DP, Botek A, et al. Health-related quality of life among patients with facial acne -- assessment of a new acne-specific questionnaire. *Clin Exp Dermatol* 2001;26(5):380-5.
24. Hornsey S, Stuart B, Muller I, et al. Patient-reported outcome measures for acne: a mixed-methods validation study (acne PROMs). *BMJ Open* 2021;11(3):e034047.
25. Ozolins M, Eady EA, Avery A, et al. Randomised controlled multiple treatment comparison to provide a cost-effectiveness rationale for the selection of antimicrobial therapy in acne. *Health Technol Assess* 2005;9(1):iii-212.

For peer review only - http://bmjopen.bmj.com/site/about/guidelines.xhtml

26. Tan JK, Tang J, Fung K, et al. Development and validation of a comprehensive acne severity scale. *J Cutan Med Surg* 2007;11(6):211-6.
27. Borm GF, Fransen J, Lemmens WA. A simple sample size formula for analysis of covariance in randomized clinical trials. *J Clin Epidemiol* 2007;60(12):1234-8.
28. Ramsey SD, Willke RJ, Glick H, et al. Cost-effectiveness analysis alongside clinical trials II-An ISPOR Good Research Practices Task Force report. *Value Health* 2015;18(2):161-72.
29. Curtis L, Burns A. *Unit Costs of Health and Social Care*. http://www.pssru.ac.uk/project-pages/unit-costs/2016/
30. Department of H. NHS reference costs 2015-16. https://www.gov.uk/government/publications/nhs-reference-costs-2015-to-2016. *Department of Health* Accessed 23 November 2017.
31. Health and Social Care Information C. Prescription Cost Analysis. http://data.gov.uk/dataset/prescription-cost-analysis-england. Accessed 23 November 2017.
32. Yang Y, Brazier J, Longworth L. EQ-5D in skin conditions: an assessment of validity and responsiveness. *Eur J Health Econ* 2015;16(9):927-39.
33. Klassen AF, Newton JN, Mallon E. Measuring quality of life in people referred for specialist care of acne: comparing generic and disease-specific measures. *J Am Acad Dermatol* 2000;43(2 Pt 1):229-33.
34. Devlin NJ, Shah KK, Feng Y, et al. Valuing health-related quality of life: An EQ-5D-5L value set for England. *Health Economics* 2018;27(1):7-22.
35. Manca A, Hawkins N, Sculpher MJ. Estimating mean QALYs in trial-based cost-effectiveness analysis: the importance of controlling for baseline utility. *Health Econ* 2005;14(5):487-96.
36. Drummond MF, Sculpger MJ, Claxton K, et al. *Methods for the economic evaluation of health care programmes.* : Oxford University Press; 2015.
37. Poinas A, Lemoigne M, Le Naour S, et al. FASCE, the benefit of spironolactone for treating acne in women: study protocol for a randomized double-blind trial. *Trials* 2020;21(1):571.

For peer review only

For peer review only - http://bmjopen.bmj.com/site/about/guidelines.xhtml

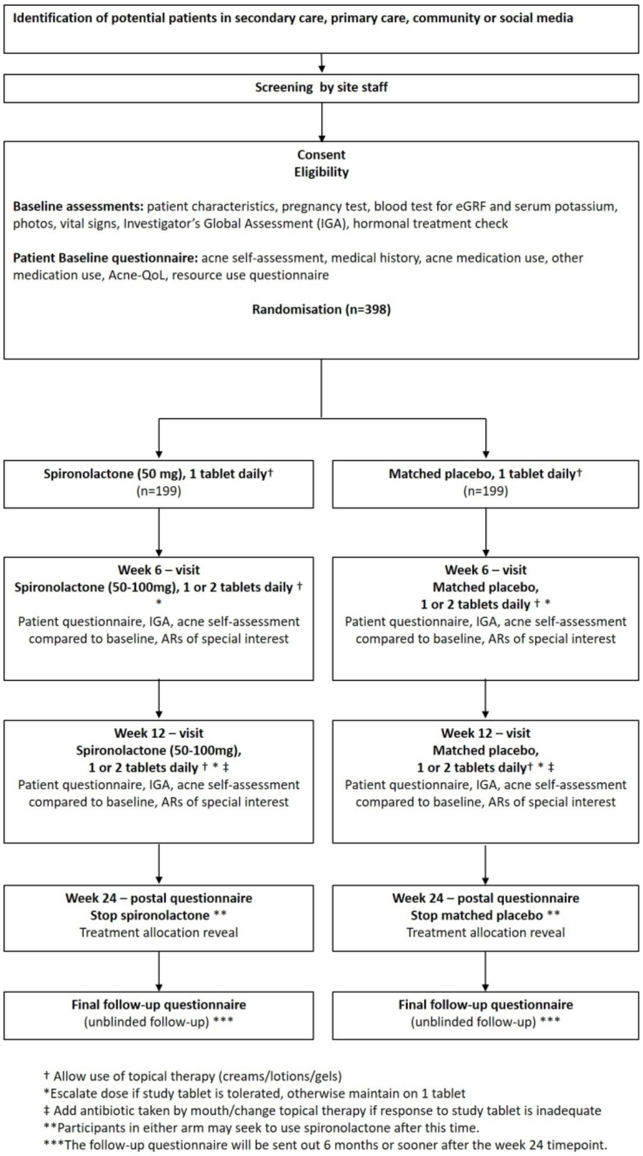

Study schema

95x177mm (300 x 300 DPI)

(Print on local headed paper)

SAFA
Spironolactone for Adult Female Acne

UNIVERSITY OF Southampton

REC Number: 18/WA/0420

IRAS ID: 246637

SAFA INFORMED CONSENT FORM

SAFA: Spironolactone for Adult Female Acne: pragmatic multicentre double-blind randomised superiority trial to investigate the clinical and cost-effectiveness of spironolactone for moderate or severe persistent acne in women

Participant Identification Number for this trial:

Name of Researcher: ______________________________

Please initial each box

1. I confirm that I have read the participant information sheet (version __ dated DD-MMM-YYYY) for the above study. I have had the opportunity to consider the information, ask questions and have had these answered satisfactorily.	INITIAL
2. I understand that my participation is voluntary and that I am free to withdraw at any time without giving any reason and without my medical care or legal rights being adversely affected.	INITIAL
3. I give permission for a copy of my consent form to be sent to the Southampton Clinical Trials Unit (where it will be stored securely), to allow confirmation of my consent.	INITIAL
4. I agree for my contact details to be shared with the Southampton Clinical Trials Unit (where stored securely), to allow the study team to contact me if needed in an emergency situation (e.g. during a pandemic).	INITIAL
5. I understand that relevant sections of my medical records, and data collected during the study, may be looked at by individuals from the Sponsor or their delegates, from Regulatory Authorities, or from the NHS Trust where it is relevant to my taking part in this research. I give permission for these individuals to have access to my records.	INITIAL
6. I understand that the information collected about me may be used to support other ethically approved research in the future, and may be shared in a pseudonymised form with other researchers.	INITIAL
7. I agree to my General Practitioner being informed of my participation in the study and to receiving information critical to my care.	INITIAL
8. I agree to not donate blood during the first 6 months of the study.	INITIAL

For peer review only

For peer review only - http://bmjopen.bmj.com/site/about/guidelines.xhtml

Funded by NIHR

(Print on local headed paper)

9. I agree to photographs of my facial acne being taken at the first clinic visit. INITIAL

10. I agree to give a blood sample at the first clinic visit. INITIAL

11. **WOMEN OF CHILD BEARING POTENTIAL AT RISK OF PREGNANCY:** INITIAL
    - I agree to take a pregnancy test at the first clinic visit
    - I agree to use my usual hormonal or barrier method of contraception as detailed in the patient information sheet
    - I agree to refrain from donation of eggs during the first 6 months of the study

12. I understand that my pseudonymised data will be held on servers located within and outside of the EU, and that access to data managed by Southampton Clinical Trials Unit (SCTU) will be strictly controlled and applicable Data Protection Legislation will be abided by. INITIAL

13. **I agree to take part in the SAFA study.** INITIAL

	YES	NO
14. **OPTIONAL**: If I withdraw from the study, I give permission for the study team to still use my pseudonymised data that has been collected.	INITIAL	INITIAL
15. **OPTIONAL:** I agree that the hospital may store photographs of my facial acne taken at my first clinic visit.	INITIAL	INITIAL
16. **OPTIONAL:** I agree to being informed of the results of the SAFA study.	INITIAL	INITIAL

Name of Participant	Signature	Date (DD-MMM-YYYY)
Name of researcher taking consent	Signature	Date (DD-MMM-YYYY)

Reminder for Research Team:

- Original signed consent form to be kept in the Investigator Site File
- 1 photocopy given to the participant
- 1 photocopy filed in the participant's medical records
- 1 scanned copy to be emailed to the Southampton CTU via secure nhs.net email account, for central monitoring purposes

For peer review only - http://bmjopen.bmj.com/site/about/guidelines.xhtml

Funded by NIHR

STANDARD PROTOCOL ITEMS: RECOMMENDATIONS FOR INTERVENTIONAL TRIALS

SPIRIT 2013 Checklist: Recommended items to address in a clinical trial protocol and related documents*

Section/item	ItemNo	Description	Adressed on page number
**Administrative information**			
Title	1	Descriptive title identifying the study design, population, interventions, and, if applicable, trial acronym	1
Trial registration	2a	Trial identifier and registry name. If not yet registered, name of intended registry	2, 17
	2b	All items from the World Health Organization Trial Registration Data Set	Included throughout manuscript
Protocol version	3	Date and version identifier	17
Funding	4	Sources and types of financial, material, and other support	17
Roles and responsibilities	5a	Names, affiliations, and roles of protocol contributors	1, 16
	5b	Name and contact information for the trial sponsor	17
	5c	Role of study sponsor and funders, if any, in study design; collection, management, analysis, and interpretation of data; writing of the report; and the decision to submit the report for publication, including whether they will have ultimate authority over any of these activities	17
	5d	Composition, roles, and responsibilities of the coordinating centre, steering committee, endpoint adjudication committee, data management team, and other individuals or groups overseeing the trial, if applicable (see Item 21a for data monitoring committee)	13
**Introduction**			

For peer review only

Background and rationale	6a	Description of research question and justification for undertaking the trial, including summary of relevant studies (published and unpublished) examining benefits and harms for each intervention	3-4
	6b	Explanation for choice of comparators	5
Objectives	7	Specific objectives or hypotheses	4
Trial design	8	Description of trial design including type of trial (eg, parallel group, crossover, factorial, single group), allocation ratio, and framework (eg, superiority, equivalence, noninferiority, exploratory)	5
**Methods: Participants, interventions, and outcomes**			
Study setting	9	Description of study settings (eg, community clinic, academic hospital) and list of countries where data will be collected. Reference to where list of study sites can be obtained	4-5
Eligibility criteria	10	Inclusion and exclusion criteria for participants. If applicable, eligibility criteria for study centres and individuals who will perform the interventions (eg, surgeons, psychotherapists)	5-7
Interventions	11a	Interventions for each group with sufficient detail to allow replication, including how and when they will be administered	7
	11b	Criteria for discontinuing or modifying allocated interventions for a given trial participant (eg, drug dose change in response to harms, participant request, or improving/worsening disease)	9, 5
	11c	Strategies to improve adherence to intervention protocols, and any procedures for monitoring adherence (eg, drug tablet return, laboratory tests)	7
	11d	Relevant concomitant care and interventions that are permitted or prohibited during the trial	5
Outcomes	12	Primary, secondary, and other outcomes, including the specific measurement variable (eg, systolic blood pressure), analysis metric (eg, change from baseline, final value, time to event), method of aggregation (eg, median, proportion), and time point for each outcome. Explanation of the clinical relevance of chosen efficacy and harm outcomes is strongly recommended	8, 9

Participant timeline	13	Time schedule of enrolment, interventions (including any run-ins and washouts), assessments, and visits for participants. A schematic diagram is highly recommended (see Figure)	7, Figure 1, Table 2
Sample size	14	Estimated number of participants needed to achieve study objectives and how it was determined, including clinical and statistical assumptions supporting any sample size calculations	9
Recruitment	15	Strategies for achieving adequate participant enrolment to reach target sample size	7, 8
**Methods: Assignment of interventions (for controlled trials)**			
Allocation:			
Sequence generation	16a	Method of generating the allocation sequence (eg, computer-generated random numbers), and list of any factors for stratification. To reduce predictability of a random sequence, details of any planned restriction (eg, blocking) should be provided in a separate document that is unavailable to those who enrol participants or assign interventions	9
Allocation concealment mechanism	16b	Mechanism of implementing the allocation sequence (eg, central telephone; sequentially numbered, opaque, sealed envelopes), describing any steps to conceal the sequence until interventions are assigned	9
Implementation	16c	Who will generate the allocation sequence, who will enrol participants, and who will assign participants to interventions	9
Blinding (masking)	17a	Who will be blinded after assignment to interventions (eg, trial participants, care providers, outcome assessors, data analysts), and how	9
	17b	If blinded, circumstances under which unblinding is permissible, and procedure for revealing a participant’s allocated intervention during the trial	9
**Methods: Data collection, management, and analysis**			

Data collection methods	18a	Plans for assessment and collection of outcome, baseline, and other trial data, including any related processes to promote data quality (eg, duplicate measurements, training of assessors) and a description of study instruments (eg, questionnaires, laboratory tests) along with their reliability and validity, if known. Reference to where data collection forms can be found, if not in the protocol	9
	18b	Plans to promote participant retention and complete follow-up, including list of any outcome data to be collected for participants who discontinue or deviate from intervention protocols	4, 7
Data management	19	Plans for data entry, coding, security, and storage, including any related processes to promote data quality (eg, double data entry; range checks for data values). Reference to where details of data management procedures can be found, if not in the protocol	10
Statistical methods	20a	Statistical methods for analysing primary and secondary outcomes. Reference to where other details of the statistical analysis plan can be found, if not in the protocol	10, 11
	20b	Methods for any additional analyses (eg, subgroup and adjusted analyses)	11, 12
	20c	Definition of analysis population relating to protocol non-adherence (eg, as randomised analysis), and any statistical methods to handle missing data (eg, multiple imputation)	10, 11
**Methods: Monitoring**			
Data monitoring	21a	Composition of data monitoring committee (DMC); summary of its role and reporting structure; statement of whether it is independent from the sponsor and competing interests; and reference to where further details about its charter can be found, if not in the protocol. Alternatively, an explanation of why a DMC is not needed	13
	21b	Description of any interim analyses and stopping guidelines, including who will have access to these interim results and make the final decision to terminate the trial	10, 11

Harms	22	Plans for collecting, assessing, reporting, and managing solicited and spontaneously reported adverse events and other unintended effects of trial interventions or trial conduct	13
Auditing	23	Frequency and procedures for auditing trial conduct, if any, and whether the process will be independent from investigators and the sponsor	13
**Ethics and dissemination**			
Research ethics approval	24	Plans for seeking research ethics committee/institutional review board (REC/IRB) approval	2, 17
Protocol amendments	25	Plans for communicating important protocol modifications (eg, changes to eligibility criteria, outcomes, analyses) to relevant parties (eg, investigators, REC/IRBs, trial participants, trial registries, journals, regulators)	17
Consent or assent	26a	Who will obtain informed consent or assent from potential trial participants or authorised surrogates, and how (see Item 32)	18, Table 1
	26b	Additional consent provisions for collection and use of participant data and biological specimens in ancillary studies, if applicable	n/a
Confidentiality	27	How personal information about potential and enrolled participants will be collected, shared, and maintained in order to protect confidentiality before, during, and after the trial	17, 18
Declaration of interests	28	Financial and other competing interests for principal investigators for the overall trial and each study site	18
Access to data	29	Statement of who will have access to the final trial dataset, and disclosure of contractual agreements that limit such access for investigators	18
Ancillary and post-trial care	30	Provisions, if any, for ancillary and post-trial care, and for compensation to those who suffer harm from trial participation	n/a

For peer review only

Dissemination policy	31a	Plans for investigators and sponsor to communicate trial results to participants, healthcare professionals, the public, and other relevant groups (eg, via publication, reporting in results databases, or other data sharing arrangements), including any publication restrictions	2
	31b	Authorship eligibility guidelines and any intended use of professional writers	16
	31c	Plans, if any, for granting public access to the full protocol, participant-level dataset, and statistical code	17
**Appendices**			
Informed consent materials	32	Model consent form and other related documentation given to participants and authorised surrogates	Additional File 2
Biological specimens	33	Plans for collection, laboratory evaluation, and storage of biological specimens for genetic or molecular analysis in the current trial and for future use in ancillary studies, if applicable	n/a

*It is strongly recommended that this checklist be read in conjunction with the SPIRIT 2013 Explanation & Elaboration for important clarification on the items. Amendments to the protocol should be tracked and dated. The SPIRIT checklist is copyrighted by the SPIRIT Group under the Creative Commons "Attribution-NonCommercial-NoDerivs 3.0 Unported" license.

For peer review only
